# Downregulation of splicing regulator RBFOX1 compromises visual depth perception

**DOI:** 10.1371/journal.pone.0200417

**Published:** 2018-07-12

**Authors:** Lei Gu, Dean Bok, Fei Yu, Joseph Caprioli, Natik Piri

**Affiliations:** 1 Department of Ophthalmology, University of California, Los Angeles, Los Angeles, CA, United States of America; 2 Department of Neurobiology, University of California, Los Angeles, Los Angeles, CA, United States of America; National Eye Centre, UNITED STATES

## Abstract

Rbfox1 is a splicing regulator that has been associated with various neurological conditions such as autism spectrum disorder, mental retardation, epilepsy, attention-deficit/hyperactivity disorder and schizophrenia. We show that in adult rodent retinas, Rbfox1 is expressed in all types of retinal ganglion cells (RGCs) and in certain subsets of amacrine cells (ACs), within the inner nuclear (INL) and ganglion cell (GCL) layers. In the INL, all Rbfox1-positive cells were colocalized with GABAergic ACs, however not all GABAergic ACs were immunostained for Rbfox1. In the GCL, a vast majority of GABAergic dACs were Rbfox1-immunopositive. Furthermore, all cholinergic starburst ACs (SACs) in the INL (type a) and in the GCL (type b) were Rbfox1 positive. The expression of Rbfox1 in the retina significantly overlapped with expression of Rbfox2, another member of Rbfox family of proteins. Rbfox2, in addition to RGCs and ACs, was also expressed in horizontal cells. In developing retinas at E12 and E15, Rbfox1 is localized to the cytoplasm of differentiating RGCs and ACs. Between P0 and P5, Rbfox1 subcellular localization switched from cytoplasmic to predominantly nuclear. Downregulation of *Rbfox1* in adult *Rbfox1*^loxP/loxP^ mice had no detectable effect on retinal gross morphology. However, the visual cliff test revealed marked abnormalities of depth perception of these animals. RNA sequencing of retinal transcriptomes of control and *Rbfox1* knockout animals identified a number of Rbfox1-regulated genes that are involved in establishing neuronal circuits and synaptic transmission, including Vamp1, Vamp2, Snap25, Trak2, and Slc1A7, suggesting the role of Rbfox1 in facilitating synaptic communications between ACs and RGCs.

## Introduction

Rbfox1 (RNA binding protein, fox-1 homolog 1) and two other members of the Rbfox family, Rbfox2 and Rbfox3, are evolutionarily conserved RNA-binding proteins that regulate tissue-specific alternative splicing. Rbfox proteins share a common domain organization and contain a single RNA recognition motif (RRM) that mediates high affinity binding to the (U)GCAUG element within alternatively spliced exons or in flanking introns.

Rbfox1 is expressed in neurons, heart, and skeletal muscle. Rbfox2 is expressed in these tissues as well as in hematopoietic and ES cells. Rbfox3 is limited to neurons; it is a well-recognized marker of post-mitotic neurons that is highly conserved among different species. Although the expression of *Rbfox* genes may overlap in most areas of the brain, their spatial pattern of expression in the cerebellar cortex, for instance, is quite different: granule cells express Rbfox1 and Rbfox3, whereas Purkinje cells express Rbfox1 and Rbfox2 [1]. The Rbfox proteins also exhibit different subcellular localization. Rbfox1 expression is observed in both the cytoplasm and nucleus of Purkinje cells, whereas Rbfox2 is restricted to the nucleus. Furthermore, the Rbfox genes exhibit distinct patterns of expression during cerebellar development. These differences in spatial and temporal expression suggest specific roles of *Rbfox* paralogs in developing and mature cerebellar neurons. *Rbfox1* neuron-specific knockout (KO) animals showed no obvious cerebellar defects but had seizures and increased neuronal excitation in the hippocampus. Whole-transcriptome analysis revealed multiple splicing changes in the *Rbfox1*−/− brain that alter proteins mediating synaptic transmission and membrane excitation [1]. Mutations, chromosomal translocations and copy number variations in human Rbfox1 have been associated with several neurodevelopmental and neuropsychiatric disorders, including autism spectrum disorder (ASD), mental retardation, epilepsy, ADHD, bipolar disorder, schizoaffective disorder and schizophrenia [2–5].

*Rbfox* genes themselves are alternatively spliced. In the case of mouse *Rbfox1*, skipping exon 19 generates a nuclear isoform with a nuclear localization signal (NLS) in the C terminus, whereas the mRNA with exon 19 encodes a protein lacking the NLS that localizes to the cytoplasm. Transcriptome profiling of mouse neurons in which *Rbfox1* was knocked out and then rescued with either nuclear or cytoplasmic isoform showed that nuclear *Rbfox1* restored splicing changes, whereas cytoplasmic *Rbfox1* rescued changes in mRNA stability and translation [6].

We first identified the expression of *Rbfox1* and *Rbfox2* in the retina when analyzing gene expression profiles of retinal ganglion cells (RGCs) [7]. In that study, to identify RGC-expressed genes we compared gene profiles of RGC-depleted and control retinas. RGC-deficient retinas were generated by optic nerve axotomy, which leads to specific RGC degeneration [7–10]. Microarray analysis was carried out with retinal RNA isolated two weeks after optic nerve transection. By that time, more than 90% of RGCs had degenerated. Genes that were underrepresented (downregulated) in RGC-deficient retinas compared to the controls, including *Rbfox1* and *Rbfox2*, were subjected to further analysis as RGC-expressed genes. From this pool of RGC-expressed genes, we had earlier identified and characterized a new RGC marker—Rbpms (RNA binding protein with multiple splicing) and Nell2 (neural epidermal growth factor-like 2) [11, 12].

Ganglion cells are the final neuronal output of the vertebrate retina; they collect visual signals from the two preceding layers of nerve cells, bipolar and amacrine cells (ACs), and transmit this information to the brain. More than 20 different types of RGCs have been identified based on their morphological characteristics such as soma size, dendritic field size, and dendritic ramification in a variety of vertebrate species. Physiologically, these cells can be classified into several major types: motion-sensitive parasol or magnocellular (M) RGCs; color-sensitive midget or parvocellular (P) RGCs that are responsible for central visual acuity; color opponent blue-yellow bistratified RGCs and RGCs responsible for pupillary reaction and for the regulation of circadian rhythm [13–17]. A recent study on functional characterization of RGCs in the mouse retina identified 32 groups of RGCs [18]. The dendritic arbors of each type of RGC cover the entire retinal surface so specific features of every point in the visual scene can be simultaneously extracted and transmitted to the brain. Degeneration of RGCs and their axons in the optic nerve causes vision loss in various optic neuropathies including its most common form, glaucoma. Glaucoma affects more than 70 million people worldwide and if left untreated, can lead to severe visual impairment and blindness (10% of total blindness cases in the US). Understanding the function of Rbfox proteins in RGCs is of particular interest since these genes play important roles during CNS neurogenesis/synaptogenesis as well as in mature neuronal function. In this study, we analyze the expression pattern of Rbfox 1 in adult and differentiating retinal neurons, evaluate its role in visual function with *Rbfox1* KO animals, and identify potential targets of Rbfox1 in RGCs and ACs by comparing retinal transcriptomes of *Rbfox1* KO and control animals.

## Results

### Expression of Rbfox1 in mature and differentiating ocular tissues

#### Rbfox1 expression in adult retinas

**Fig 1:**
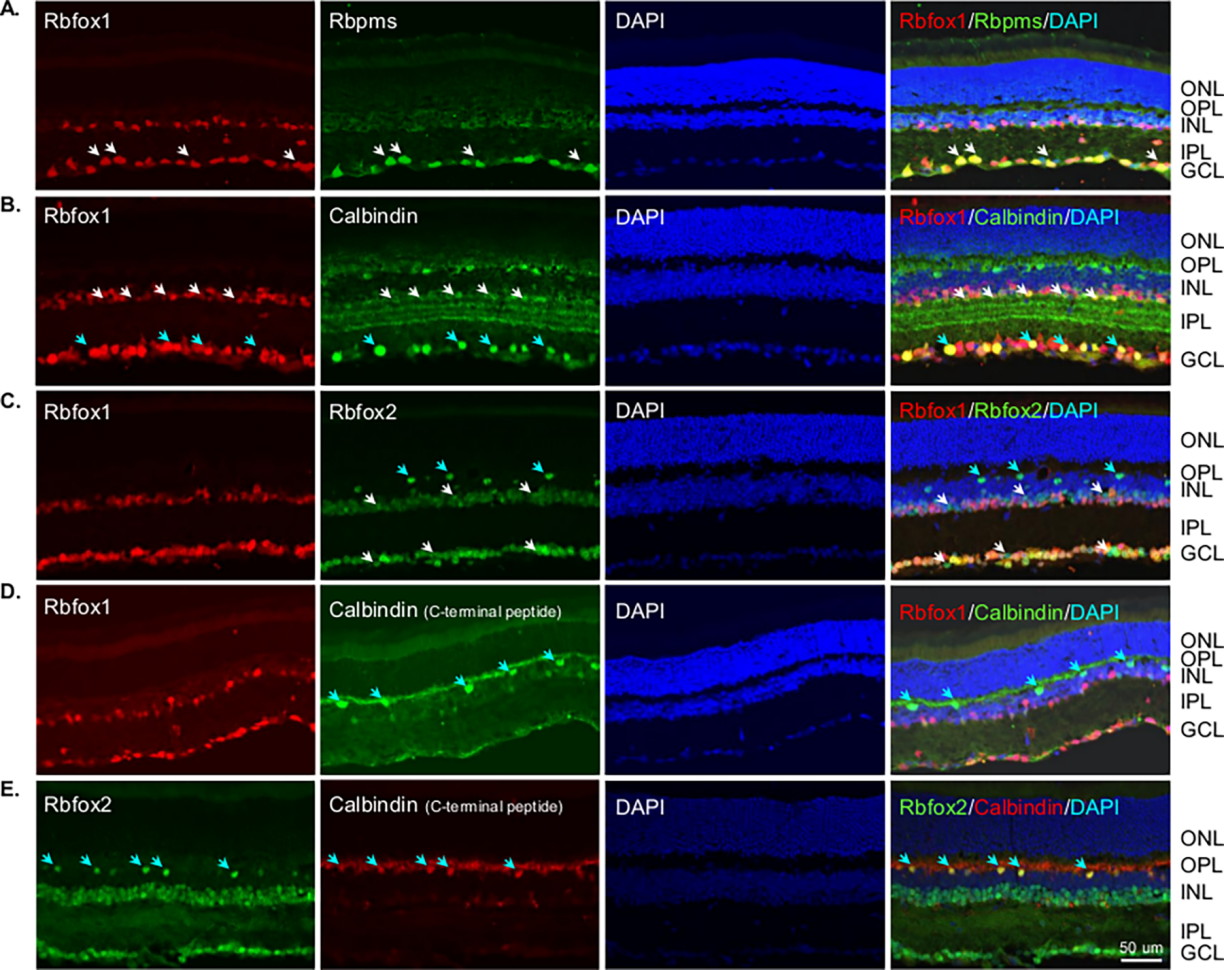
Immunohistochemical localization of Rbfox1 expression in mouse retinal sections. Rbfox1 immunoreactivity is present in the ganglion cell layer (GCL) and inner nuclear layer (INL) of the retina. Rbfox1-positive cells in the INL, which contains cell bodies of horizontal cells (HCs), bipolar cells and amacrine cells (ACs), as well as Muller glia cells, were primarily localized proximal to the inner plexiform layer (IPL). A. Rbfox1 was colocalized with Rbpms-positive RGCs. White arrows point at several Rbfox1/Rbpms-positive RGCs. B. Calbindin-positive displaced ACs (dACs; blue arrows) and ACs with cell bodies localized at the margin with IPL (white arrows) were also immunoreactive for Rbfox1. C. Colocalization of Rbfox1 and Rbfox2 showed significant overlap in expression of these genes within GCL. In the INL, as stated above, Rbfox1 is expressed in ACs adjacent to the IPL, whereas the expression of Rbfox2 is more widely distributed among ACs. White arrows point at Rbfox2-positive/Rbfox1-negative ACs and dACs in the INL and GCL, respectively. Blue arrows indicate Rbfox2-positive HCs. D and E. Colocalization of Rbfox1 and Rbfox2 with cells that are immunoreactive for calbindin generated against C-terminal peptide. These anti-calbindin antibodies show strong immunoreactivity in HCs. Arrows point at HCs in the INL that are immunoreactive for calbindin and Rbfox2. ONL, outer nuclear layer, OPL; outer plexiform layer, DAPI; 4',6-diamidino-2-phenylindole.

**Fig 2:**
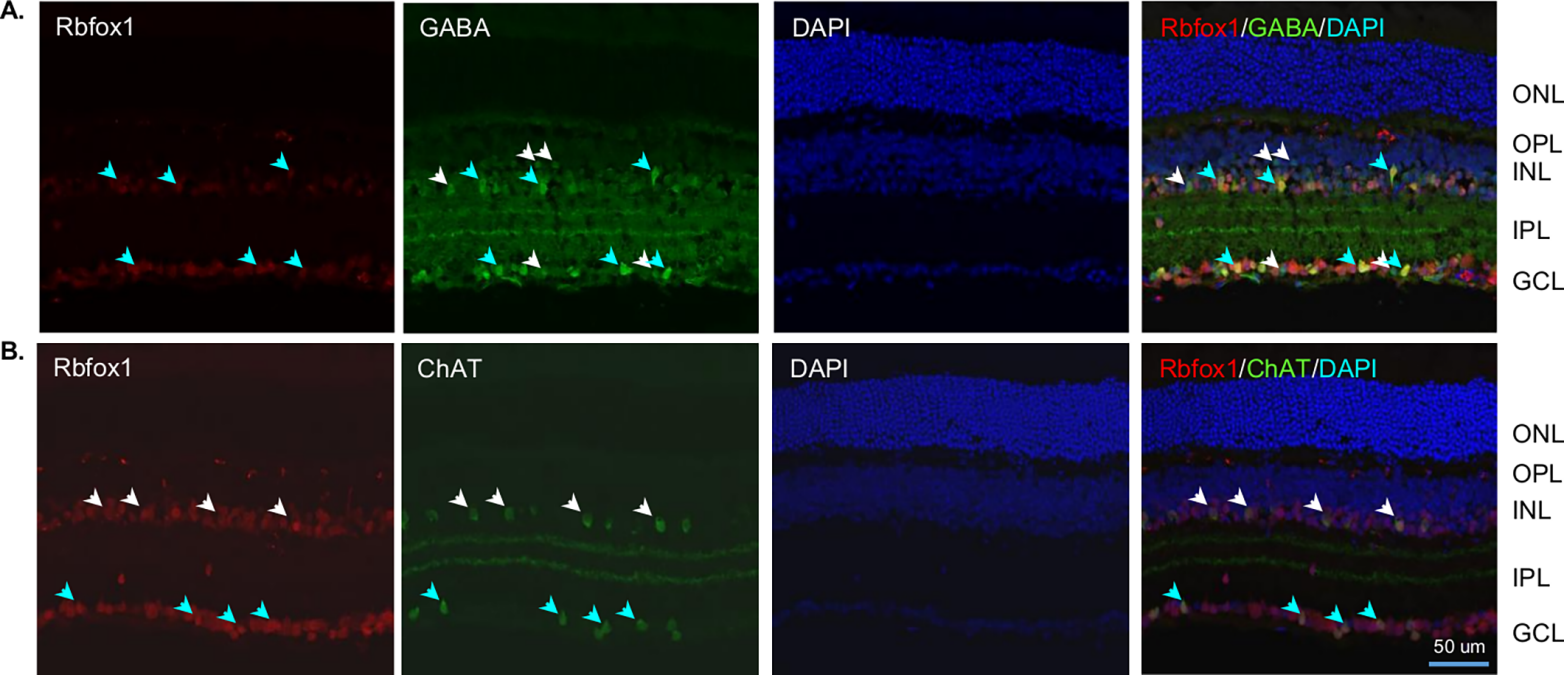
Immunohistochemical colocalization of Rbfox1 positive cells with GABAergic and cholinergic amacrine cells. A. In the inner nuclear layer (INL) many Rbfox1 immunoreactive cells were colocalized with GABAergic ACs. Blue arrows point at several Rbfox1/GABA-positive cells in the INL. White arrows point at GABAergic cells in the INL and GCL that were not immunostained for Rbfox1. In the ganglion cell layer (GCL), a vast majority of GABAergic dACs were Rbfox1-immunopositive. B. All ChAT-immunoreactive starburst ACs (SACs) in the INL (type a) and in the GCL (type b) were Rbfox1-positive. White and blue arrows point at several type a and type b SACs, respectively that are colocalized with Rbfox1-positive cells. ONL, outer nuclear layer, OPL, outer plexiform layer; IPL, the inner plexiform layer; DAPI (4',6-diamidino-2-phenylindole).

**Fig 3:**
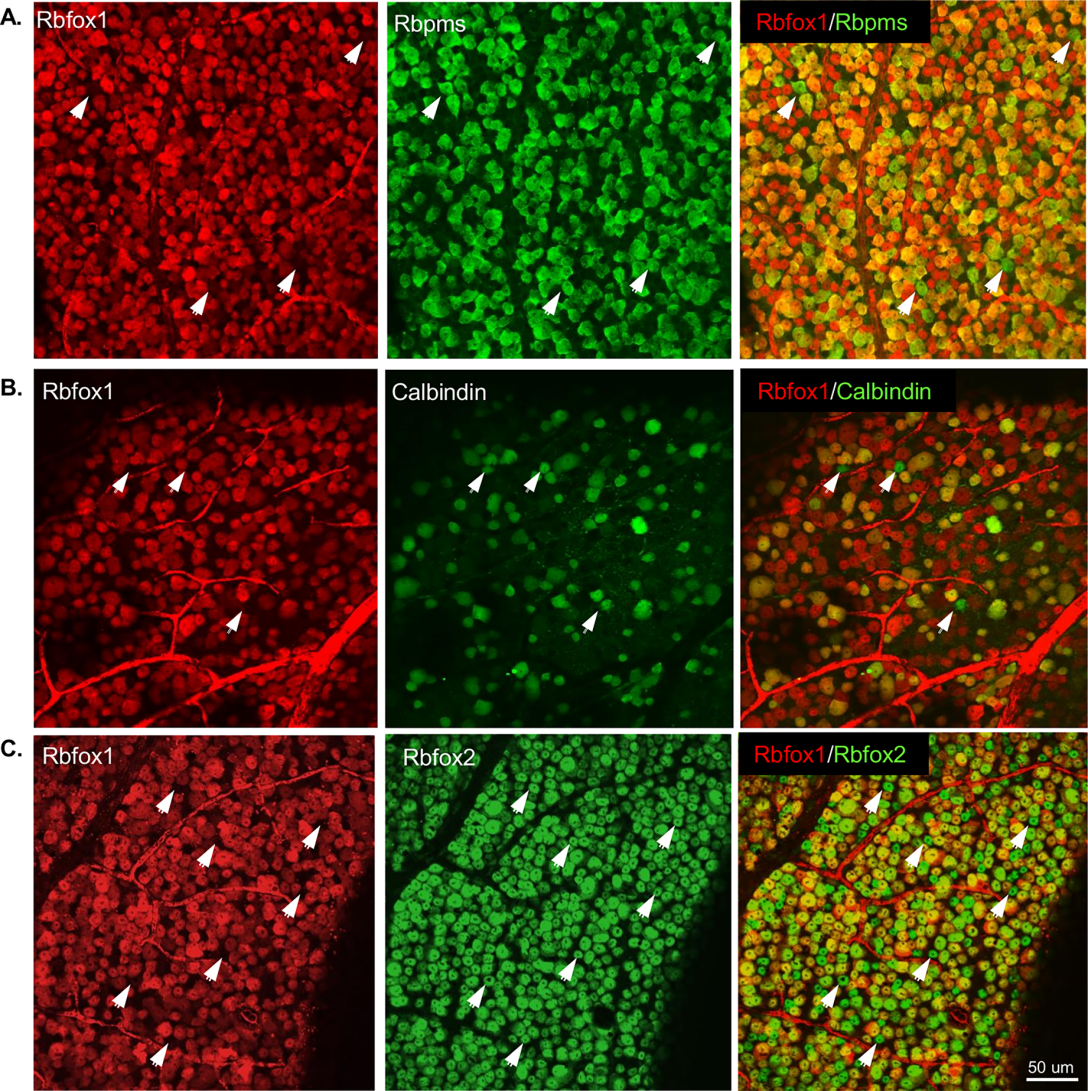
Quantitative analysis of Rbfox1-positive cells in whole mount adult mouse retina. A. All Rbpms-positive RGCs were also stained with Rbfox1 antibody. There are some Rbpms stained cells that appeared to be negative for Rbfox1 (indicated by arrowheads), but at higher magnification these cells have faint Rbfox1 staining. B. Approximately 94% of calbindin-positive cells were also positive for Rbfox1. C. Colocalization of Rbfox1 and Rbfox2 positive cells revealed approximately 6% of cells with Rbfox2 expression were negative for Rbfox1. Arrowheads in (B) and (C) point at cells immunoreactive for calbindin or Rbfox2, respectively but negative for Rbfox1.

We first characterized the spatial expression pattern of Rbfox 1 in adult mouse retinas (Fig 1). Results of the immunohistochemistry with anti-Rbfox1 antibodies revealed predominant localization of Rbfox1 expression in the ganglion cell layer (GCL) and inner nuclear layer (INL) of the retina. The GCL in rodent retinas contains two types of neurons, RGCs and displaced ACs (dACs), in a ratio of approximately 1:1. Rbfox1 expressing cells in the INL, which contains cell bodies of horizontal cells (HCs), bipolar cells and ACs, as well as Muller glia cells, were primarily localized proximal to the inner plexiform layer. ACs of the mouse retina form two to four rows of cells at the inner margin of the INL [19, 20], which suggests that Rbfox1-positive cells in the INL are ACs. We used Rbpms as a marker for RGCs [11] and calbindin (immunogen—purified bovine cerebellum calbindin D-28K protein) to identify ACs, dACs and horizontal cells (HCs), although ACs in the INL and dACs in the GCL show variable expression of this marker [21, 22]. As seen in Fig 1A, all Rbpms-positive RGCs also express Rbfox1. Calbindin-labeled dACs and some ACs with cell bodies localized at the margin with inner plexiform layer (IPL) were also positive for Rbfox1 (Fig 1B). Colocalization of Rbfox1 and Rbfox2 showed significant overlap in expression of these genes in cells within the GCL (Fig 1C). In the INL, as stated above, Rbfox1 was mostly expressed in ACs that were adjacent to the IPL, whereas Rbfox2 expression was more broadly distributed among ACs. Furthermore, Rbfox2 expression was also observed in HCs: images in Fig 1D and 1E show that calbindin-positive (immunogen—C-terminal peptide of rat calbindin D-28K) HCs express Rbfox2, but not Rbfox1. Although calbindin is used as a marker for both ACs and HCs in the mouse retina, anti-calbindin antibodies generated against C-terminal peptide appear to label HCs much stronger than ACs [22, 23]. To further define the subtype identity of Rbfox1-positive ACs, retinal sections were immunostained for Rbfox1 along with gamma-aminobutyric acid (GABA) and choline acetyltransferase (Chat) neurotransmitters. GABA is expressed by medium to large-field types of ACs, including cholinergic starburst cells (SACs). Most, if not all, Rbfox1-positive cells in the INL were colocalized with GABAergic ACs, whereas not all GABAergic ACs were immunostained with Rbfox1 (Fig 2A). In the GCL, the vast majority of GABAergic dACs were Rbfox1-immunopositive. Rbfox1-positive cells in the GCL that do not express GABA are most likely RGCs (RGCs typically do not express GABA) or glycinergic dACs. All ChAT-immunoreactive SACs in the INL (type a) and in the GCL (type b) were Rbfox1 positive (Fig 2B). Quantitative analysis of Rbfox1-expressing cells in the GCL was performed by counting Rbfox1/Rbpms, Rbfox1/calbindin and Rbfox1/Rbfox2 positive cells. Cells were counted in the whole mounted retinas 0.5 mm from the center of the optic nerve in the superior, inferior, nasal and temporal quadrants (Fig 3). Virtually 100% of RGCs (Rbpms-positive cells) were positive for Rbfox1 (3644 ± 104.44 cells/mm^2^; Fig 3A). However, among calbindin-positive cells (2513 ± 45.92 cells/mm^2^) a small population of cells–approximately 6.2%—had no Rbfox1 staining, suggesting that some subtype(s) of dACs do not express this protein (2358 ± 47.34 Rbfox1/calbindin-positive cells/mm^2^; Fig 3B). This was further supported by colocalization of Rbfox1 and Rbfox2 positive cells (Fig 3C). Rbfox2, like Rbfox1, was expressed in all RGCs, but approximately 6.4% of cells with Rbfox2 expression were negative for Rbfox1 (6487 ± 108.79 cells/mm^2^ Rbfox2-positive and 6069 ± 109.76 cells/mm^2^ Rbfox1/Rbfox2-positive). Since ~100% of RGCs are positive for both Rbfox1 and Rbfox2, these 6.4% of Rbfox2-positive cells are dACs, suggesting that in both GCL and INL (Fig 1C), Rbfox1 expression among dACs and ACs, respectively is restricted to fewer subtypes of these cells compared to Rbfox2.

#### Rbfox1 expression during retina development

**Fig 4:**
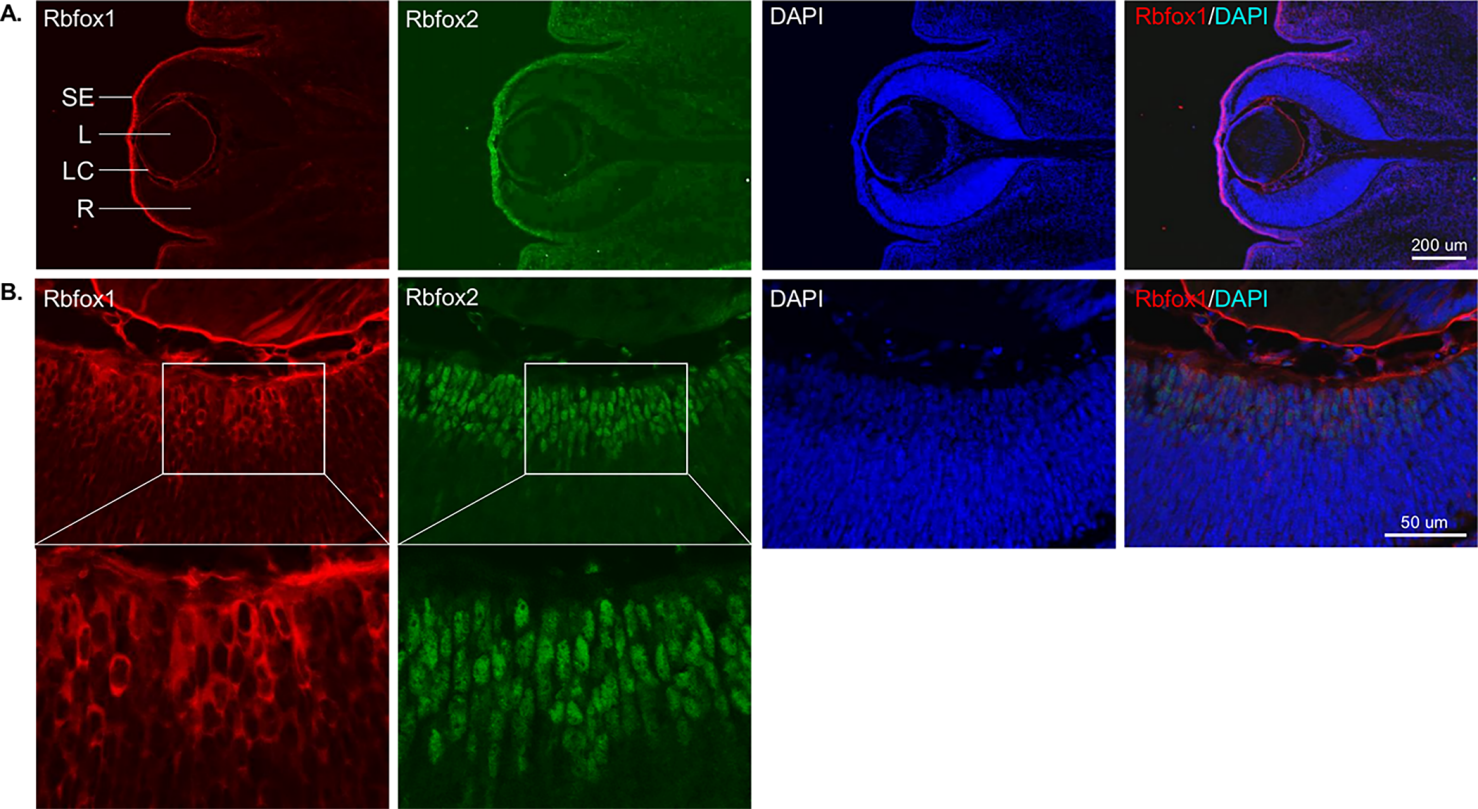
Rbfox1 expression pattern at embryonic day 12 (E12) of mouse ocular development. A. A distinctive Rbfox1 staining at E12 is clearly present in the surface ectoderm, lens and retina. B. Rbfox1 expression in retinal cells appears to be cytoplasmic, whereas, Rbfox2 expression is predominantly nuclear. The boxed area in is enlarged below. These cells are most likely RGCs, since at that developmental stage RGCs are the main type of differentiated retinal cells. L, lens; LC, lens capsule; R, retina; SE, surface ectoderm.

**Fig 5:**
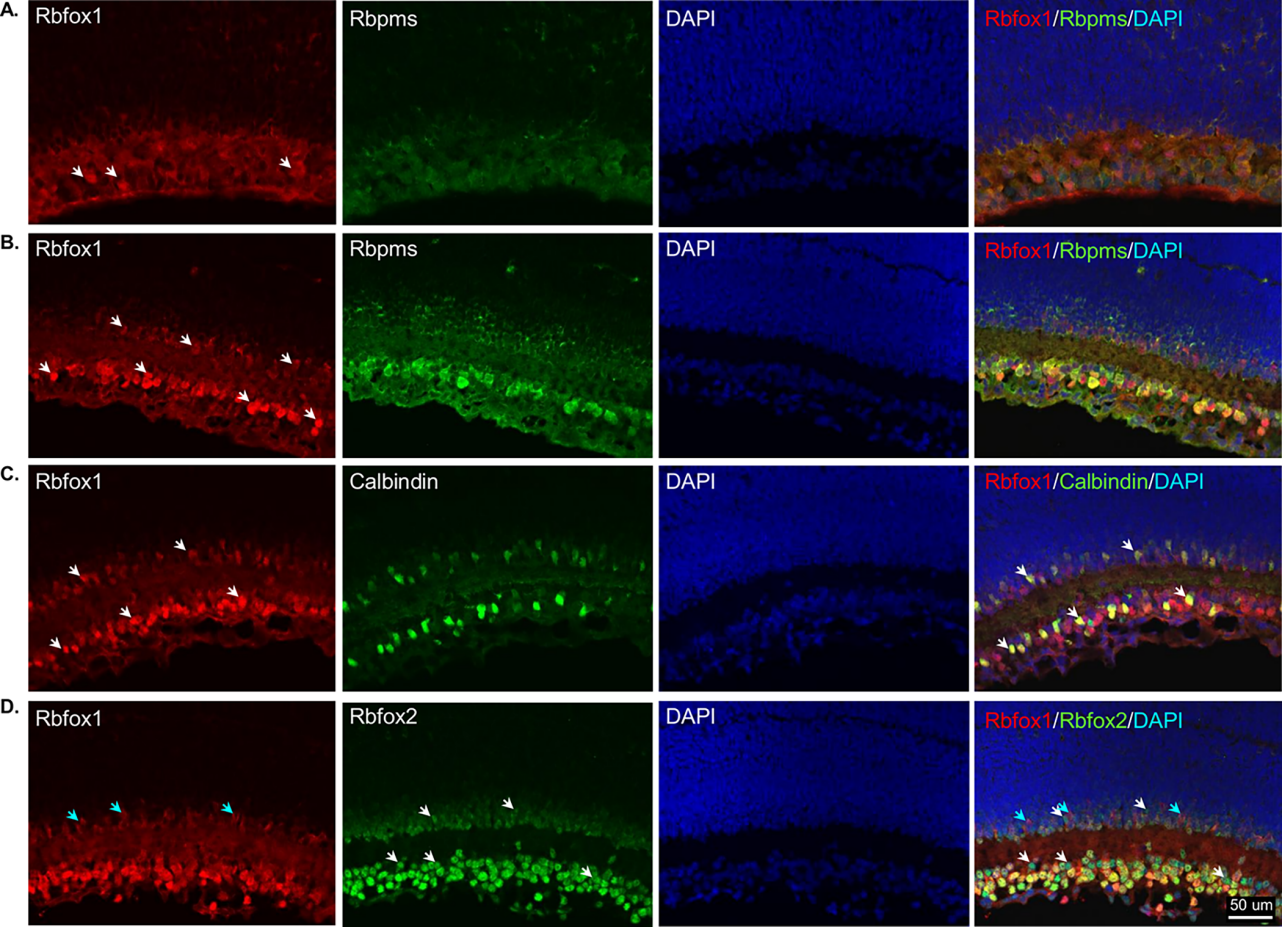
Dynamics of subcellular localization of Rbfox1 during early postnatal development of the retina. A. At P0, along with cells with cytoplasmic Rbfox1 expression, there are cells in the GCL with pronounced nuclear staining (pointed by arrows). Most of Rbfox1 and Rbpms expression is colocalized indicating that these cells are RGCs. B and C. At P5, Rbfox1 expression switches to be predominantly nuclear especially in RGCs and dACs. More cells with nuclear expression of Rbfox1 also appear in the INL. Arrows point at several cells in the GCL and INL with Rbfox1 nuclear staining. D. A significant overlap exists between Rbfox1 and Rbfox2 expression at P5, though in both, the GCL and INL, there are cells with specific expression of one or the other paralog. Rbfox1 staining in the IPL (B, C and D) is non-specific since it was also observed in a control experiment with only secondary antibodies. Blue and white arrows point at several Rbfox1-positive/Rbfox2-negative and Rbfox2-positive/Rbfox1-negative cells, respectively.

Since Rbfox1 is involved in neurogenesis and synaptogenesis, we analyzed its expression pattern during differentiation of retinal cells starting from embryonic day 12 (E12). The temporal order of retinal cells generation from multipotent progenitor cells is conserved among many species. In mice, RGCs and HCs differentiate first, followed in overlapping phases by cone-photoreceptors, ACs, rod photoreceptors, bipolar cells and, finally, Müller glia cells [24]. RGCs are born between E8 and E17, with a peak around E11; ACs are born between E8 and P5, with a peak around E16 [20]. Rbfox1 staining at E12 was present in the retina, cornea and lens (Fig 4A). Rbfox1 expression in retinal cells appeared to be cytoplasmic, whereas Rbfox2 expression was predominantly nuclear (Fig 4B). These Rbfox1/Rbfox2-positive cells were most likely RGCs, since at that developmental stage RGCs are the main type of differentiated retinal cells. There was no remarkable change in the pattern of Rbfox1 expression at E15 compared to E12. At P0, however, along with cells with cytoplasmic Rbfox1 expression, there were cells in the GCL with nuclear Rbfox1 staining (Fig 5A). At this age, we were able to identify Rbpms-positive RGCs, which in general were positive for Rbfox1. At P5, Rbfox1 expression becomes predominantly nuclear especially in RGCs and dACs (Fig 5B and 5C). Rbfox1 staining in the IPL (Fig 5B, 5C and 5D), which contains dendrites of RGCs and ACs, is non-specific since it was also observed in a control experiment without Rbfox1 primary antibodies. Rbfox1 and Rbfox2 expression showed a significant overlap, though in both, the GCL and INL, there were cells with specific expression of one or the other paralog (Fig 5D). Rbfox1, as well as of Rbfox2, expression patterns at P10, P12, P14, P15 and P21 were very similar to that of adult animals shown in Fig 1.

#### Rbfox1 and Rbfox2 staining in the lens and cornea

**Fig 6:**
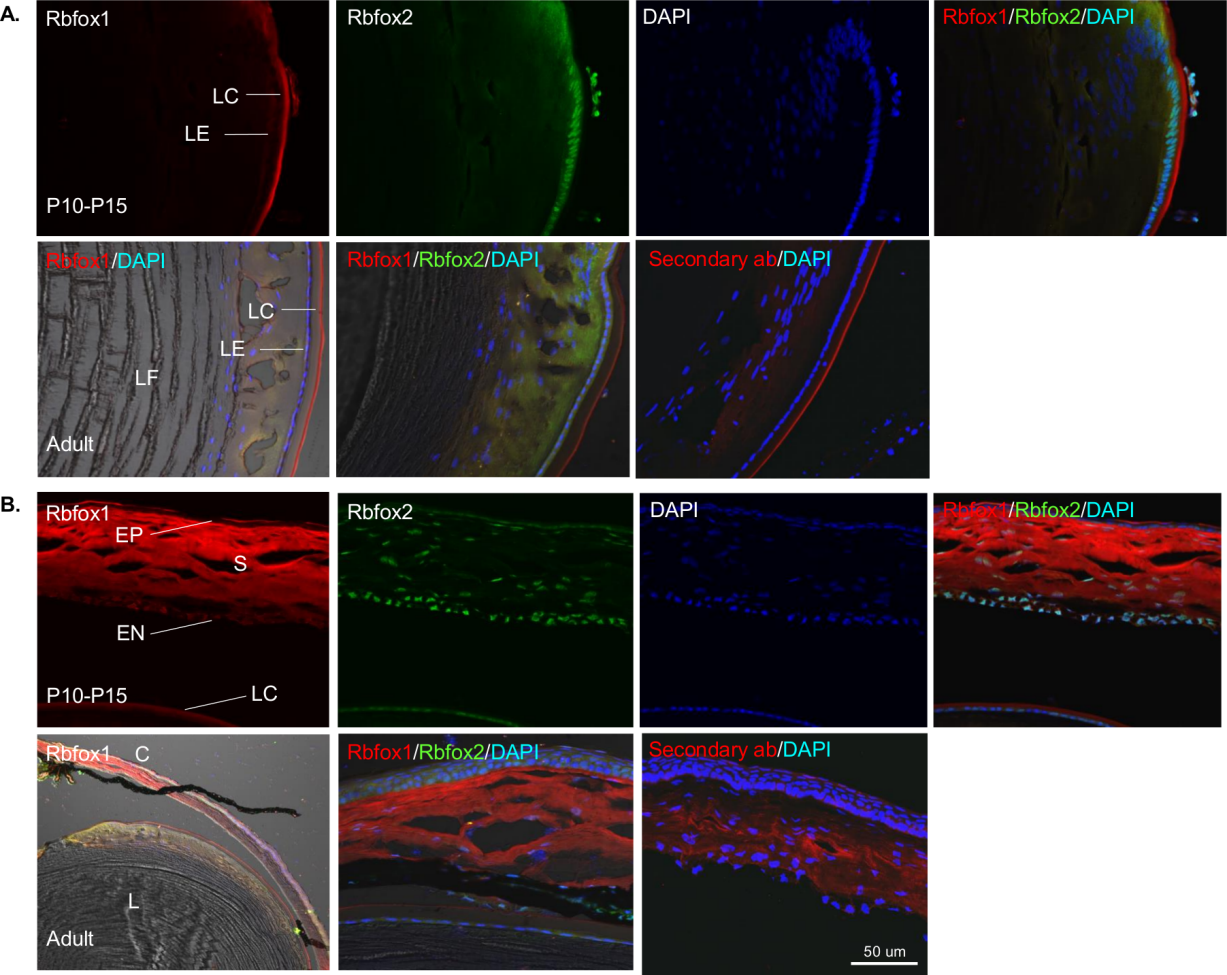
Rbfox1 distribution in the lens and cornea. A. Rbfox1 staining in the lens observed at E12 (Fig 3A) remained unchanged at P10, P12, P14, P15 and in adult animals. It was predominantly localized in the lens capsule (non-specific), although relatively faint expression can be clearly seen in the cytoplasm of lens epithelial cells from P10 throughout adulthood. Rbfox2 expression is localized to lens epithelial cells (appears to be nuclear) and fiber cells. B. In the cornea, strong Rbfox1 staining is observed in the stroma (non-specific) and endothelial cells. Less pronounced expression is present in epithelial cells. The pattern of this staining in the cornea did not undergo detectable change from P10 into adult stage. Rbfox2 expression was observed in corneal epithelial and endothelial cells, as well as keratocytes in the stroma. Strong Rbfox1 staining in lens capsule and corneal stroma is non-specific since it is present in negative control immunohistochemistry without Rbfox1 primary antibodies.

Distinctive Rbfox1 staining was present in the lens and in the surface ectoderm in the developing mouse eye at E12 (Fig 4A). The Rbfox1 staining pattern remained unchanged in the lens at P10, P12, P14, P15 and in adult animals. It was predominantly localized to the lens capsule. Relatively faint expression can be seen in the cytoplasm of lens epithelial cells from P10 throughout adulthood (Fig 6A). Rbfox2 expression was primarily localized to lens epithelial cells and fiber cells. With respect to the cornea, strong Rbfox1 staining was observed in the stroma and endothelial cells. Less pronounced expression was present in epithelial cells. The pattern of this staining in the cornea did not undergo any detectable change from P10 into the adult stage (Fig 6B). Expression of Rbfox2 was observed in the corneal epithelial and endothelial cells, as well as in keratocytes. Rbfox2 in both lens and corneal cells was predominantly localized to cell nuclei. The Rbfox1 staining in the extracellular matrix of lens capsule and corneal stroma was also present in a control experiment without Rbfox1 primary antibodies indicating that the observed reaction was not specific to Rbfox1 (Fig 6A and 6B).

### *Rbfox1* KO mice have depth perception deficiency with no detectable change in gross retinal morphology

**Fig 7:**
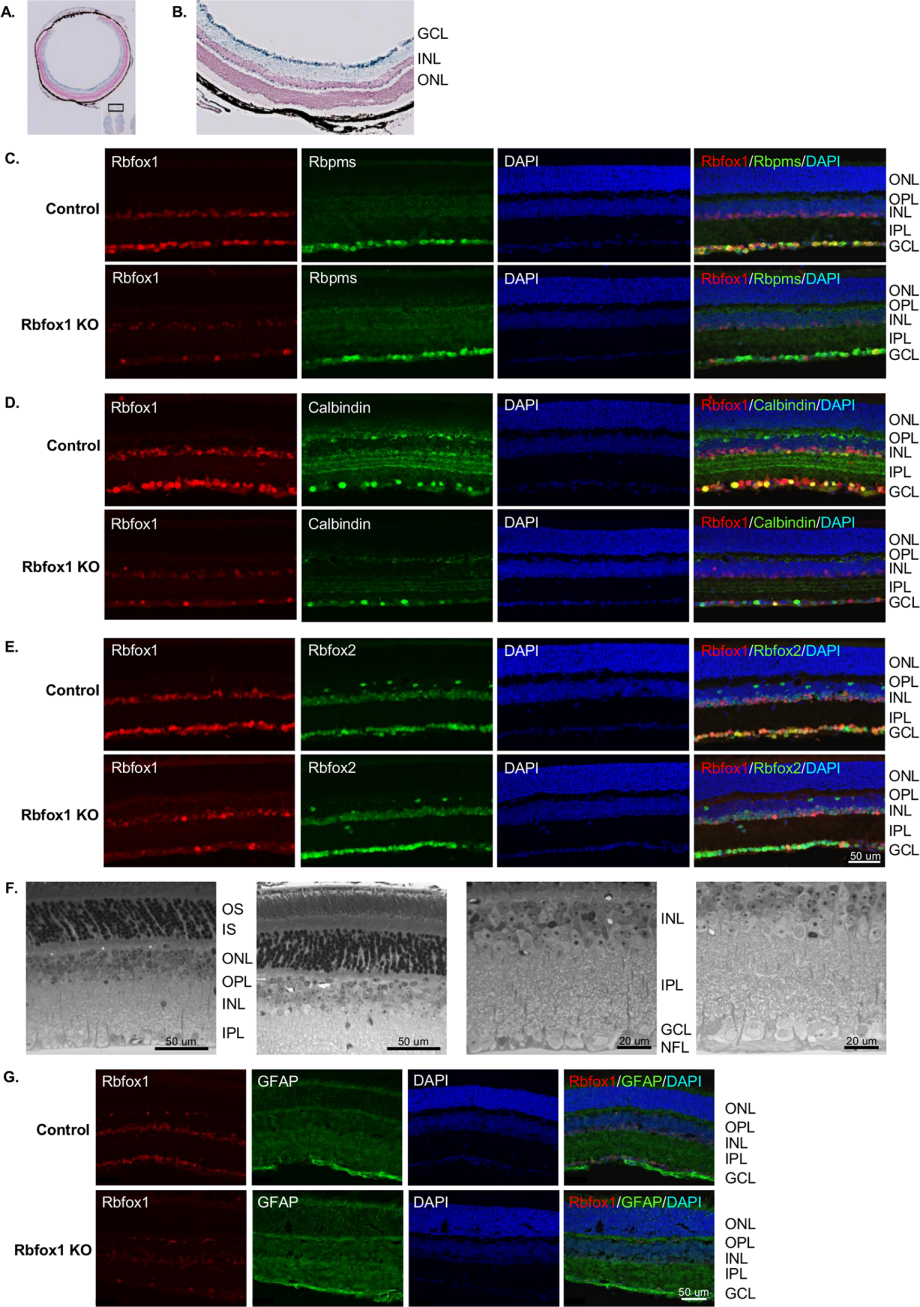
*Rbfox1* KO animals have normal retinal morphology. A and B. The Tg(UBC-cre/ERT2)1Ejb transgenic mouse was used to generate Rbfox1 KO animals. The images (modified from www.informatics.jax.org/recombinase/specificity?id=MGI:3707333&system=sensory+organs) show beta-galactosidase expression in ocular tissues of an adult Tg(UBC-cre/ERT2)1Ejb transgenic mouse. The rationale for using this transgene is based on a strong Cre expression in RGCs, optic nerve, in the majority of dACs, and certain types of ACs adjacent to the IPL, as well as in the cornea. These locations are most relevant to study the effect of Rbfox1 downregulation on visual function. C-E. Colocalization of Rbfox1 with Rbpms (C), calbindin (D) and Rbfox2 (E) in retinas of wild-type and Rbfox1 KO animals. Very few Rbfox1-positive cells in the GCL were detected. Less dramatic (compared to the GCL), but evident downregulation of Rbfox1 is also observed among ACs in the INL. D. Calbindin immunoreactivity is decreased in Rbfox1 KO retinas; almost no calbindin staining is observed in the INL and very few calbindin-positive cells are present in the GCL. E. Rbfox2 expression in the GCL cells appears to be increased. F. Light microscopic examination of epoxy resin-embedded specimens also failed to identify any change in retinal morphology two months after Rbfox1 downregulation. G. Expression of glial fibrillary acidic protein (GFAP) in *Rbfox1* KO retinas. GFAP staining was used to evaluate potential “stress” in the retina associated with Rbfox1 downregulation. However, no significant difference in the levels of GFAP immunoreactivity between control and *Rbfox1* KO retinas was observed. OS, photoreceptor outer segments; IS, photoreceptor inner segments; ONL, outer nuclear layer; OPL, outer plexiform layer; INL, inner nuclear layer; IPL, inner plexiform layer; GCL, ganglion cell layer.

*Rbfox1* KO animals were generated by crossing mice carrying conditional *Rbfox1* alleles, *Rbfox1*^loxP/loxP^ [1], with mice carrying a Cre-ERT2 fusion gene controlled by the human UBC promoter sequence [UBC-Cre^+/−^; Tg(UBC-cre/ERT2)1Ejb]. The UBC-Cre-ERT2 mouse was used in these experiments since it shows robust Cre expression in RGCs, in the majority of dACs, and in ACs adjacent to the IPL (Fig 7A and 7B; modified from www.informatics.jax.org/recombinase/specificity?id=MGI:3707333&system=sensory+organs) [25]. In addition to the cells that are relevant for this study, Cre expression in the retina was also present in some sparsely distributed cells in the INL adjacent to the OPL, presumably HCs. Furthermore, expression of Cre was observed in the cornea. Cre recombinase expression was induced in adult (2 months old) homozygous *Rbfox1*^loxP/loxP^; UBC-Cre^+/−^ animals by daily (for 5 days) administration of tamoxifen. Experimental animals were viable, had normal growth rate and behaviorally had no apparent anomalies compared to control animals.

#### *Rbfox1* downregulation has no effect on retinal architecture

**Fig 8:**
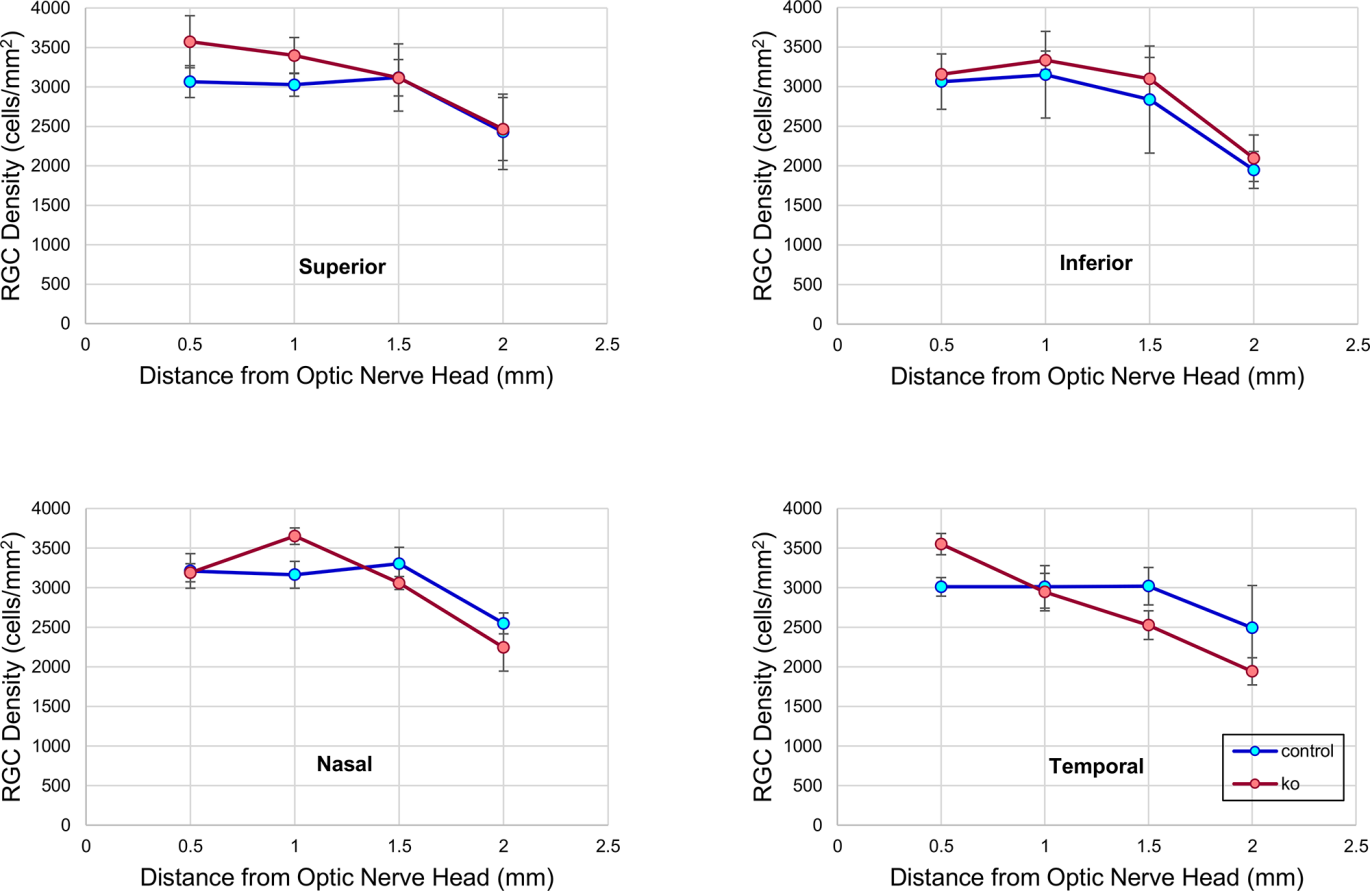
RGC quantification in retinas of *Rbfox1* KO animals. The possible effect of Rbfox1 downregulation on RGC survival was evaluated two months after last administration of tamoxifen. RGCs were immunolabeled with antibodies against Rbpms and counted in inferior, superior, temporal and nasal retinal quadrants. The average densities of RGCs in all four retinal quadrants of Rbfox1 KO (n = 3) and control (n = 3) animals were similar: in the superior quadrant 2913 ± 338.14 cells/mm^2^ in control vs 3139 ± 364.21 cells/mm^2^ in Rbfox1 KO, P = 0.376; in the inferior quadrant 2750 ± 502.14 cells/mm^2^ in control vs 2921 ± 341.66 cells/mm^2^ in *Rbfox1* KO, P = 0.526; in the nasal quadrant 3057 ± 238.79 cells/mm^2^ in control vs 3037 ± 339.50 cells/mm^2^ in *Rbfox1* KO, P = 0.898; and in the temporal retina 2884 ± 310.62 cells/mm^2^ in control vs 2743 ± 387.54 cells/mm^2^ in *Rbfox1* KO, P = 0.461. Data are presented as the mean ± SEM.

Two months after the last tamoxifen administration, *Rbfox1* KO animals were analyzed to determine the extent of *Rbfox1* downregulation and its effect on retinal architecture, particularly on RGC morphology and survival. Immunohistochemistry shows a dramatic reduction of Rbfox1 expression in cells within the GCL (Fig 7C, 7D and 7E). Very few Rbfox1-positive cells in this retinal layer were detected; only one Rbfox1-stained RGC was present on the representative image (Fig 7C). Less dramatic (compared to the GCL) but strong downregulation of Rbfox1 was also observed among ACs in the INL. We also noted a marked decrease in calbindin immunereactivity in *Rbfox1* KO retinas; almost no calbindin staining was observed in the INL and very few calbindin-positive cells were present in the GCL (Fig 7D). Interestingly, calbindin immunorectivity in *Rbfox1* KO retinas was also decreased in HCs that normally do not appear to be immunoreactive for Rbfox1. It is possible, however, that the level of Rbfox1 expression in these cells is below the level of immunohistochemical detection used in this study to evaluate expression of this gene in retinal cells. Calbindin is one of the major Ca-binding proteins that maintain Ca homeostasis by buffering excessive intracellular levels of free Ca and its downregulation in certain types of dACs and ACs may alter their normal function. With respect to Rbfox2, it appeared that its expression in the GCL is somewhat increased in experimental compared to control animals’ retinas (Fig 7E). The observed strong downregulation of Rbfox1 in the GCL and relatively weak downregulation in the INL of *Rbfox1* KO animals reflects the Cre expression pattern in Tg(UBC-cre/ERT2)1Ejb mouse retina (Fig 7A and 7B). Overall, Rbfox1 KO retinal gross morphology looked normal; no obvious abnormalities in cellular architecture were detected. EM examination also failed to identify any change in retinal morphology two months after Rbfox1 downregulation (Fig 7F). Furthermore, *Rbfox1* KO retinas were immunostained for glial fibrillary acidic protein (GFAP), a commonly used indicator of "stress", which in the retina, is upregulated primarily in the activated Müller glial cells [26]. We observed no significant difference in the levels of GFAP immunoreactivity between control and *Rbfox1* KO retinas (Fig 7G). To determine whether *Rbfox1* downregulation has any effect on RGC numbers, RGC quantification was performed in the superior, inferior, nasal and temporal retinal quadrants (Fig 8). RGC density in any of the four retinal quadrants were similar between control and *Rbfox1* KO retinas: in the superior quadrant 2913 ± 338.14 cells/mm^2^ in control vs 3139 ± 364.21 cells/mm^2^ in Rbfox1 KO, P = 0.376; in the inferior quadrant 2750 ± 502.14 cells/mm^2^ in control vs 2921 ± 341.66 cells/mm^2^ in *Rbfox1* KO, P = 0.526; in the nasal quadrant 3057 ± 238.79 cells/mm^2^ in control vs 3037 ± 339.50 cells/mm^2^ in *Rbfox1* KO, P = 0.898; and in the temporal retina 2884 ± 310.62 cells/mm^2^ in control vs 2743 ± 387.54 cells/mm^2^ in *Rbfox1* KO, P = 0.461. Retinas from three control and three Rbfox1 KO animals used for RGC quantification.

#### Depth perception deficiency in *Rbfox1* KO mice

**Fig 9:**
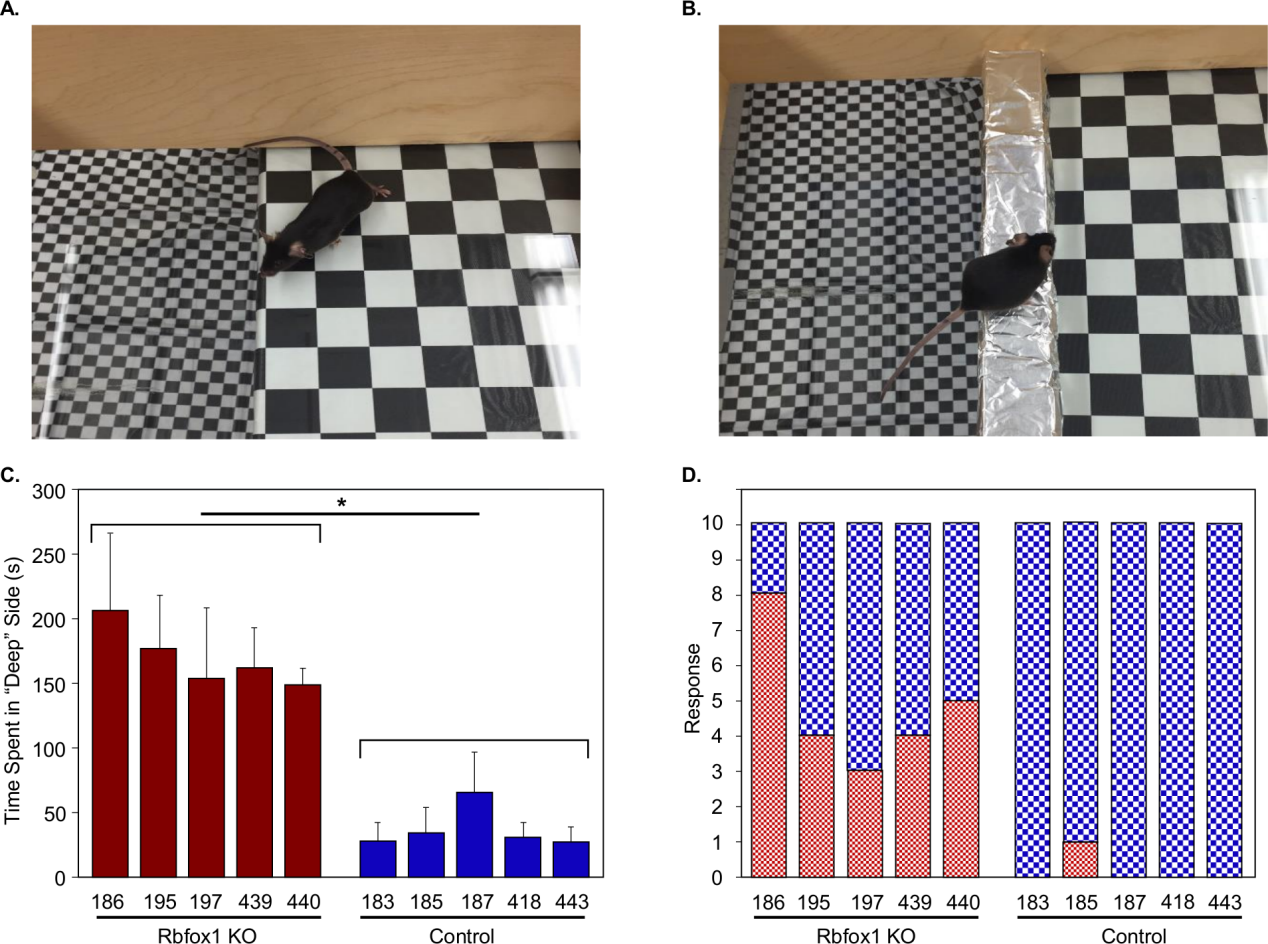
Visual cliff test reveals depth perception impairment in Rbfox1 KO mice. Rbfox1 KO and control mice were subjected to two modifications of the test: A. the first test determines the time the animal spends in deep versus shallow side of the chamber; B. in the second test animals were placed on a pedestal between deep and shallow sides and animal’s preference to step down on the perceived deep or shallow side was recorded. The test is designed to identify visual dysfunction that can alter animal’s avoidance of the deep side of the chamber. C. *Rbfox1* KO mice spent more time on the deep side than on the shallow side. The overall mean (+/-SD) time spent on the deep side was 179.7 +/- 58.8 seconds for all animals in the *Rbfox1* KO group and 42.3 +/- 28.8 seconds for all animals in the control group, respectively. There was a statistically significant mean difference in time spent on the deep side between two groups: 137.4 seconds (95% CI = 82.8–192.0 seconds; p = 0.002). D. *Rbfox1* KO mice were also less selective when choosing shallow vs deep side to step down. Control mice have clear preference for the shallow side of the box and avoid the deep side (C and D).

We evaluated visual behavior in *Rbfox1* null mice by subjecting them to the visual cliff test, which has been shown to be effective in distinguishing between animals with normal and poor visual abilities [27]. The test relies on the innate tendency of animals to avoid the deep side of a visual cliff field. Two modifications of the test were performed: the first determined the time the animal spends in the deep versus shallow side of the chamber (Fig 9A) and the second determined the animal’s preference for the shallow or perceived deep side when it was placed on a pedestal between deep and shallow sides (Fig 9B). Both tests showed that control mice have a clear preference for the shallow side of the box and avoid the deep side (Fig 9C and 9D). *Rbfox1* KO animals, on the other hand, spent more time on the deep side than on the shallow side. The overall mean (+/- SD) time spent on the deep side was 179.7 +/- 58.8 seconds for all animals in the *Rbfox1* KO group and 42.3 +/- 28.8 seconds for all animals in the control group, respectively. There was a statistically significant mean difference in time spent on the deep side between two groups: 137.4 seconds (95% CI = 82.8–192.0 seconds; p = 0.002). When choosing the side to step down, shallow or deep, the preference for the shallow side among all *Rbfox1* KO animals was 50% compared to 97% among all animals in control group. The odds ratio (OR) of preference for the shallow side between two groups was statistically significantly less pronounced among *Rbfox1* KO animals than control animals: 0.034 (95% CI = 0.005–0.24; p = 0.042).

#### *Rbfox1* KO retinal transcriptome analysis

**Fig 10:**
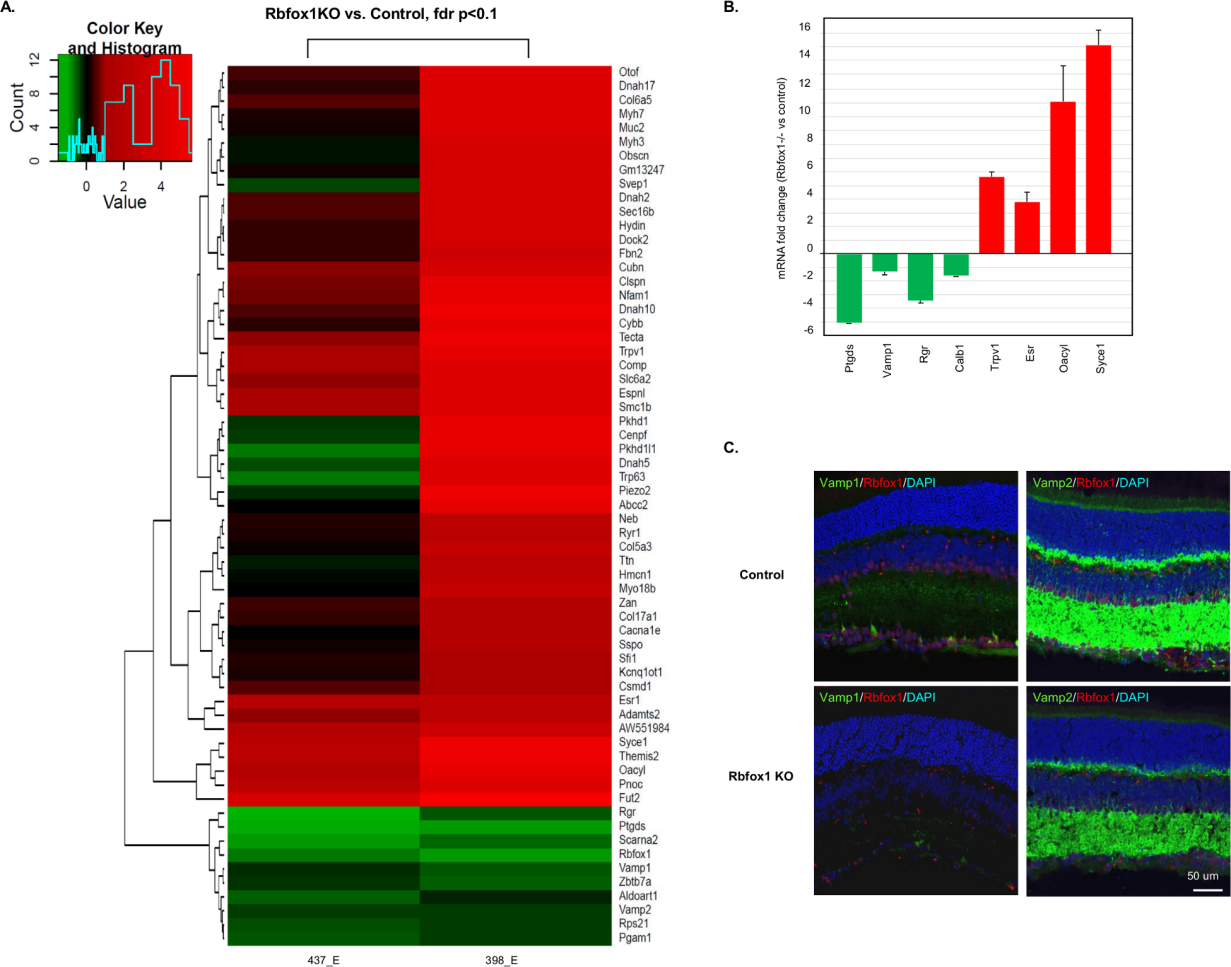
Identification of Rbfox1-regulated genes in the retina. A. Heatmap representing the top differentially expressed genes in *Rbfox1* KO vs control retinas after RNA sequencing. Rows are transcripts and each column is an experimental sample compared to three control samples. Red and green in the heat map indicate up- and down-regulation, respectively. B. Real-time PCR quantification of several differentially regulated genes identified by RNA-seq. Downregulation of prostaglandin D2 synthase (*Ptgds*), vesicle-associated membrane protein 1 (*Vamp1*), RPE-retinal G protein receptor (*Rgr*), calbindin 1 (*Calb1*) and upregulation of transient receptor potential cation channel, subfamily V, member 1 (*Trpv1*), estrogen receptor 1 (*Esr*), O-acyltransferase like protein (*Oacyl*) and synaptonemal complex central element protein 1 (*Syce1*) in the retinas of *Rbfox1* KO animals observed by quantitative real-time PCR are in agreement with the results of RNA-seq. The data are presented as the mean ± SEM. C. Immunohistochemical analysis of Vamp1 and Vamp2 in mouse retinal sections. Vamp1 is localized to a very small population of RGCs, whereas Vamp2 is widely expressed in the inner and outer plexiform layers. Vamp1 and Vamp2 were noticeably downregulated in retinas of *Rbfox1* KO animals, which is consistent with RNA-seq and real-time PCR data. ONL, outer nuclear layer; OPL, outer plexiform layer; INL, inner nuclear layer; IPL, inner plexiform layer; GCL, ganglion cell layer.

A robust expression of Rbfox1 in both ACs and RGCs suggests its role in regulation of genes involved in the functions of these cells and possibly their communications. To test this hypothesis, we performed RNA sequencing on *Rbfox1* KO and control animals. RNAs with altered alternative splicing or expression level were analyzed to identify Rbfox1 targets within ACs and RGCs as well as potential candidates responsible for visual dysfunction in Rbfox1 null animals. Our list of top rated differentially expressed genes in *Rbfox1* KO retinas was dominated by 53 upregulated genes; only 10 transcripts are downregulated, including Rbfox1 (Fig 10A). Real-time PCR analysis of several up- and downregulated genes was used to confirm the RNA-seq data (Fig 9B). We also picked a couple of genes (Vamp1 and Vamp2; vesicle-associated membrane proteins) for immunohistochemical analysis to show that the spatial localization of differentially regulated genes identified by RNA-seq is relevant to the Rbfox1 expression pattern in the retina (Fig 10C). Vamp2 shows abundant staining in the IPL (contains synaptic connections between RGCs, ACs and bipolar cells) and outer plexiform layer (OPL; contains synaptic connections between photoreceptors, bipolar and horizontal cells), whereas Vamp1 is restricted to a very few RGC somas and their dendrites (Fig 10C). The observed Vamp1 and Vamp2 staining pattern in control retinas is very similar to that reported earlier [28]. Vamp1 and Vamp2 immunoreactivity is visibly reduced in Rbfox1 KO compared to control retinas, which further corroborates RNA-seq and real-time PCR data on Rbfox1-mediated expression of these genes in the retina (Fig 10C). Top rated genes with differential expression levels and genes with differential splicing patterns that have been shown to have neuronal functions or be associated with different neurological conditions are listed in Tables 1 and 2, respectively. Additional information on Rbfox1-regulated genes, including a complete gene list including fold changes (experimental vs. control), uncorrected and FDR corrected p-values, and absolute gene level quantification using FPKM and log count per million (logCPM; S1 Table) and a list of top rated differentially spliced genes in Rbfox1 KO animals (S1 Fig), is presented in Supplementary Information.

**Table 1:** Differentially regulated genes in *Rbfox1* null mouse retinas that have been reported to be have neuronal function.

Gene	Regulation
Esr1, estrogen receptor 1 (alpha)	+
Otof, otoferlin	+
Ptgds, prostaglandin D2 synthase	-
Slc6a2, solute carrier family 6 (neurotransmitter transporter), member 2	+
Trp63, transformation related protein 63	+
Trpv1, transient receptor potential cation channel, subfamily V, member 1	+
Vamp1, vesicle-associated membrane protein 1	-
Vamp2, vesicle-associated membrane protein 2	-
Arpc3, actin related protein 2/3 complex, subunit 3	DS
Add1, adducin 1 (alpha)	DS
Cacna1a, Calcium Voltage-Gated Channel Subunit Alpha1 A	DS
Cntn1, contactin 1	DS
Epb41l1, erythrocyte membrane protein band 4.1 like 1	DS
Eps8, epidermal growth factor receptor pathway substrate 8	DS
Kptn, kaptin	DS
Kdr, kinase insert domain protein receptor	DS
Mdm2, transformed mouse 3T3 cell double minute 2	DS
Mpdz, multiple PDZ domain protein	DS
Nedd4, neural precursor cell expressed, developmentally down-regulated 4	DS
Nptn, neuroplastin	DS
Rufy3, RUN and FYVE domain containing 3	DS
Slc1a7, solute carrier family 1 (glutamate transporter), member 7	DS
Snap25, synaptosomal-associated protein 25	DS
Trak2, trafficking protein, kinesin binding 2	DS

“+” and “-”indicate up- or downregulation, respectively; DS, differentially spliced

**Table 2:** Association of differentially regulated genes in retinas of Rbfox KO animals with neurological diseases.

Gene	Gene-associated disease	Regulation
Cenpf, Centromere Protein F	Stromme syndrome, Apple peel syndrome with microcephaly and ocular anomalies, Jejunal atresia with microcephaly and ocular anomalies	+
Comp, Cartilage Oligomeric Matrix Protein	Alzheimer's disease (AD), Parkinson's disease (PD), Huntington's disease (HD), Creutzfeldt-Jacob disease (CJD), Gerstmann-Straussler disease (GSD), Gerstmann-Straussler-Scheinker disease (GSSD), Fatal familial insomnia, Glycine encephalopathy	+
Hmcn1, Hemicentin 1	Age-related macular degeneration	+
Neb, Nebulin	Distal myopathy, Nemaline myopathy, Mohr-Tranebjaerg syndrome	+
Oacyl, O-acyltransferase like protein	Canavan disease, Charcot-Marie-Tooth disease, Progressive myoclonic epilepsy, Distal hereditary motor neuropathies, Pontocerebellar hypoplasia, Spastic ataxia	+
Otof, Otoferlin	Deafness, autosomal recessive	+
Ptgds, ProstaglandinD2 synthase	Bipolar disorder	-
Tecta, Tectorin Alpha	Bilateral sudden sensorineural hearing loss, Deafness, autosomal recessive and autosomal dominant	+
Vamp1, Vesicle Associated Membrane Protein 1	Spastic ataxia	-
Zan, Zonadhesin	Hereditary spastic paraplegia	+
BBS2, Bardet-Biedl Syndrome 2	Bardet-Biedl syndrome	DS
Cacna1A, Calcium Voltage-Gated Channel Subunit Alpha1 A	Dominantly inherited ataxias or spinocerebellar ataxias, Early infantile epileptic encephalopathy, Episodic ataxias, Hemiconvulsion-hemiplegia-epilepsy syndrome	DS
Epb41L1, Erythrocyte Membrane Protein Band 4.1 Like 1	Autosomal dominant mental retardation	DS
Gle1, Gle1 RNA Export Mediator	Lethal arthrogryposis with anterior horn cell disease	DS
Kptn, kaptin	Autosomal recessive mental retardation	DS
Kcnma1, Potassium Calcium-Activated Channel Subfamily M Alpha 1	Generalized epilepsy and paroxysmal dyskinesia	DS
Mdm2, Mdm2 Proto-Oncogene	Glioma, Retinoblastoma, Medulloblastoma	DS
Sf1, Splicing Factor 1	Multiple sclerosis	DS
Tsen34, tRNA Splicing Endonuclease Subunit 34	Pontocerebellar hypoplasia	DS
Ttl, Tubulin Tyrosine Ligase	Niemann-Pick disease type C, Oculopharyngeal muscular dystrophy, Nonsyndromic autosomal recessive mental retardation, Progressive myoclonic epilepsy, Leber congenital amaurosis, Myoclonic Epilepsy and Ragged-Red Fiber Disease	DS
Wdr36, WD Repeat Domain 36	Primary open angle glaucoma	DS
Zdhhc1, Zinc Finger DHHC-Type Containing 1	Syndromic X-linked mental retardation with epilepsy or seizures	DS

“+” and “-”indicate up- or downregulation, respectively; DS, differentially spliced

## Discussion

The first evidence for Rbfox1 expression in the retina we observed when analyzing gene expression profiles of RGCs [7]. To identify RGC-expressed genes in that study, we compared gene profiles of wild-type adult rat retinas with RGC-depleted retinas from animals two weeks after optic nerve axotomy. In the current study, we investigated the Rbfox1 expression pattern in developing and adult rodent retina, and evaluated the effect of its downregulation on visual function. We showed that Rbfox1 expression in the fully differentiated retina is restricted to RGCs and dACs in the GCL and certain subtypes of ACs in the INL. Quantitative analysis indicates that virtually 100% of RGCs, irrespective of their morphological and functional differences (RGCs in the mouse retina are represented by more than 32 distinct functional types), are positive for Rbfox1. Rbfox1 was also expressed in vast majority of dACs; some calbindin- or Rbfox2-positive cells in the GCL were negative for Rbfox1. Most, if not all, Rbfox1-positive cells in the INL were colocalized with GABAergic ACs, and in the GCL, the vast majority of GABAergic dACs were Rbfox1-immunopositive. Furthermore, all ChAT-immunoreactive SACs in the INL (type a) and in the GCL (type b) were Rbfox1-positive. Rbfox1 expression in the INL appears to be restricted to ACs that are located in the innermost layer of the INL, whereas Rbfox2-expressing cells are present in several rows occupied by ACs. Approximately 30 AC subtypes have been identified in mammalian retinas and somas of distinct AC subtypes have been shown to differ in their position in the INL and GCL; for example, within the INL, somas of most ChAT^+^ and NPY^+^ ACs are located adjacent to the IPL, whereas somas of Ebf^+^ ACs are closer to the outer plexiform layer [20]. This suggests that cell-specific expression of Rbfox1 and Rbfox 2 may be important for the differentiation and function of distinct AC subtypes.

Rbfox1 expression during ocular development was analyzed at E12, E15, P0, P5, P10, P12, P14, P15 and P21. The rationale for choosing these time points was based on the timeline of retinal cell differentiation and establishment of synaptic connections [20, 29]. We observed Rbfox1 cytoplasmic staining in differentiated RGCs between E12 and P0. The shift from cytoplasmic to predominantly nuclear localization started at P0 with very few cells and was more prominent at P5. By P10, Rbfox1 distribution in RGCs and ACs is predominantly nuclear and remained as such in the fully differentiated retina. It has been reported that chronic depolarization of neurons leads to alternative splicing of Rbfox1 transcripts, resulting in the increased expression of the splicing-active nuclear Rbfox1 isoform [30]. The shift in Rbfox1 subcellular distribution coincides with the stage II spontaneous retinal waves of excitation, which in mice begins around the time of birth and lasts the first 1–2 postnatal weeks. Generated by cholinergic synaptic transmission between SACs and from SACs to RGCs, stage II waves are critical for the refinement of visual circuits in a frequency- and pattern-dependent manner [31–33]. Unlike Rbfox1, expression of Rbfox2 in both RGCs and ACs was consistently nuclear at all stages of retinal development (from E12 to P21), and in the mature retina. Dynamic regulation of Rbfox1 subcellular localization suggests different roles of this protein during retinal differentiation.

To understand the role of Rbfox1 in adult retinas, we have evaluated the effect of Rbfox1 downregulation on retinal morphology and function, as well as compared transcriptional profiles of *Rbfox1* KO and control retinas to identify Rbfox1 regulated genes. No changes in retinal morphology or in RGC density two month after Rbfox1 downregulation were observed. To evaluate the role of Rbfox1 on visual function, the visual cliff test was used. The test evaluates the functional integrity of the retino-geniculo-cortical pathway by determining the depth perception of the animal [27, 34]. The test is based on the animals’ innate tendency to avoid the deep side of the chamber. In this study, we utilized two modifications of the cliff test: first, we determined the time the animal spent on the shallow versus perceived deep side and second, we determined the animal’s preference for the shallow versus deep side to step down from the platform placed between the sides. In both cases, as expected, control animals showed strong avoidance of the illusionary deep side. *Rbfox1* KO mice on the other hand, spent more time on the deep side than on the shallow side and were less selective in choosing the shallow side when stepping down from the platform. The test clearly identifies depth perception impairment caused by downregulation of the *Rbfox1* gene. The exact location(s) of the defects in the visual pathway is difficult to pinpoint, since downregulation of *Rbfox1* in *Rbfox1*^loxP/loxP^/UBC-Cre^+/-^ animals may take place not only in the retina but also in various brain structures that receive and process visual information. To identify Rbfox1-regulated genes, including those that may be associated with depth perception deficiency in *Rbfox1* KO animals, retinal transcriptomes of control and *Rbfox1*-deficient animals were analyzed for alternatively spliced as well as differentially expressed genes. Among the relatively small number of top rated differentially regulated genes, there were at least 35 genes that have been shown to have neuron-specific functions or associated with various neurological diseases, including Vamp1, Vamp2 and Snap25 (synaptosomal-associated protein 25). These proteins together with syntaxins and synaptotagmin form the core of the SNARE [soluble *N*-ethylmaleimide-sensitive factor (NSF) attachment protein (SNAP) receptors] complex that mediates synaptic vesicle fusion [35].

We were rather surprised to see Rgr (RPE-retinal G protein receptor) among the top 10 downregulated genes. Rgr is an opsin expressed in the RPE and in the end-feet of Müller glial cells. It was first described as a putative photoisomerase [36] but later was shown to regulate the traffic of retinyl esters in RPE cells by modulation of retinyl ester hydrolase (REH) and lecithin retinyl acyl transferase [37]. However, at least two publications show Rgr expression in RGCs [38, 39]. It was suggested that Rgr plays a role in retinoid photoisomerization in Opn4-expressing intrinsically photosensitive RGCs [39]. Interestingly, the expression pattern of Rgr appears to be relatively even across the GCL and its level of expression on immunoblot was comparable to the protein isolated from highly enriched preparations of photoreceptor cells and retinal pigment epithelium. Considering the fact that the ipRGCs constitute a very small subpopulation of RGCs (<5%), Rgr’s role in the GCL may go beyond retinoid photoisomerization in ipRGCs.

In summary, Rbfox1 is expressed in all RGC types and in certain types of ACs in the GCL and INL. There is a significant overlap between Rbfox1 and Rbfox2 expression, however, in both the GCL and INL there are cells with specific expression of one or the other paralog. In the developing retina, Rbfox1 is present as early as E12. Rbfox1 cytoplasmic localization in RGCs and ACs between E12 and P0 shifts to predominantly nuclear expression, starting at P0 with very few cells, and was more prominent at P5. By P10, Rbfox1 distribution in RGCs and ACs is predominantly nuclear and remains as such in fully differentiated retina. The shift in Rbfox1 subcellular distribution can be correlated with the stage II spontaneous retinal waves of excitation, which in mice takes place during first 1–2 postnatal weeks. To gain insights into Rbfox1 function in the retina, *Rbfox1* KO animals were generated. Although no morphological abnormalities were observed in the retinas of adult *Rbfox1* KO mice 2 months after downregulation, these animals have an evident deficiency in depth perception. Transcriptome analysis of control and *Rbfox1* KO retinas identified several proteins with known function in synaptic transmission, including Vamp1, Vamp2, Snap25, Trak2, and Slc1A7, suggesting a role of Rbfox1 in regulation of genes involved in establishing and maintaining the proper neural circuits formed by RGCs and ACs.

## Experimental procedures

### Animals

The use of animals and all experimental procedures with animals were approved by the Animal Research Committee of the University of California at Los Angeles and were performed in compliance with the National Institutes of Health Guide for the Care and Use of Animals and the ARVO (The Association for Research in Vision and Ophthalmology) Statement for the Use of Animals in Ophthalmic and Vision Research. *Rbfox1* KO animals were generated by crossing homozygous transgenic mice with loxP sites flanking *Rbfox1* gene exons 11–12 (*Rbfox1*^loxP/loxP^; kindly provided by Dr. Douglas Black, UCLA [1]) with Tg(UBC-cre/ERT2)1Ejb mice carrying the Cre recombinase gene driven by the human ubiquitin C (UBC) promoter (Jackson Laboratory, Bar Harbor, ME; [25]), which express tamoxifen-inducible Cre. The resulting heterozygous Rbfox1^loxP/+;^ UBC-Cre^+/-^ mice were crossed to *Rbfox1*^loxP/loxP^ mice to obtain homozygous *Rbfox1*^loxP/loxP^/UBC-Cre^+/-^ animals. Mice used in this study were maintained on the C57BL/6J background. Age-matched heterozygous Rbfox1^loxP/+^ mice were used as controls. Animals were housed in a 12-h light-dark cycle with food and water available ad libitum. Carbon dioxide (CO_2_)-induced asphyxiation was used to euthanize animals.

### Induction of Cre activity with tamoxifen

Tamoxifen (T5648; Sigma, St. Louis, MO) was dissolved in corn oil (Sigma) at 37°C for 30 min to a final concentration of 50 mg/ml. Tamoxifen solutions were freshly prepared prior to oral gavage. Mice were administered 200 mg/kg body weight of tamoxifen solution or corn oil (vehicle) every 24 hours, for a total of 5 doses. Recipient mice were housed individually to avoid tamoxifen cross-contamination [40].

### Immunohistochemistry of the retina and other ocular tissues

Eyes were enucleated, fixed with ice-cold 4% paraformaldehyde and cryoprotected in 30% sucrose. 14-μm thick retinal sections were cut with cryostat. Sections were blocked for 30 min with blocking solution (20% fetal calf serum, 5% goat serum, 0.1% Triton X-100 in PBS) and incubated with primary antibodies at 4°C overnight. Sections were then washed three times with 0.1% Triton X-100 in PBS and stained with secondary antibodies for 1 hour at room temperature. Sections were again washed three times, mounted with mounting medium containing DAPI reagent and imaged using a confocal laser scanning microscope Olympus FV3000 (Olympus, MA). The following primary antibodies were used: anti-Rbfox1 produced in mouse, 1:200 (Novus Biologicals, Littleton, CO); anti-Rbfox2 produced in rabbit, 1:1500 (Bethyl Laboratories, Montgomery, TX); anti-Rbpms produced in rabbit, 1:500 [11]; anti-Rbpms produced in guinea pig, 1:1000 (kindly provided by Dr. Nicholas Brecha, UCLA); anti-calbindin D-28K produced in rabbit, 1:500 (EMD Millipore, Billerica, MA); anti-calbindin D-28K produced in rabbit, 1:500 (C2724, Sigma); anti-GABA produced in rabbit, 1:2000 (Sigma); anti-ChAT produced in goat, 1:200 (EMD Millipore); anti-GFAP produced in rabbit, 1:400 (Sigma). The following secondary antibodies were used: Alexa Fluor 488-conjugated goat anti-rabbit IgG, 1:500; Alexa Fluor 568-conjugated goat anti-mouse IgG, 1:100; and Alexa Fluor 568-conjugated goat-anti-guinea pig IgG, 1:100 (Thermo Fisher Scientific, Canoga Park, CA).

### Processing of mouse retinal tissue for light microscopy

After isofluorane anesthesia and cardiac perfusion with 2% formaldehyde and 2.5% glutaraldehyde in 0.1M phosphate buffer, pH 7.2. E, eyes were enucleated and placed in 1% osmium tetroxide in 0.1M phosphate buffer for 1 h. After rinsing three times in cold distilled water (4°C), the eyes were dehydrated in a series of cold (4°C) ethanol (50, 70, 95, and 100%) and infiltrated with propylene oxide three times for 10 min each and then with 1:2 and 2:1 of araldite/propylene oxide for 30 min each on a rotator. The eyes were placed in pure araldite for 1 h and then embedded in flat embedding molds at the 60°C for 48 h. Once the blocks were fully polymerized, 1 μm retinal sections were cut with a Leica Ultracut UCT ultramicrotome. Sections were collected on glass slides and stained with 1% toluidine blue in 1% sodium borate, then photographed with a Zeiss Axiophot microscope fitted with a Planapo 20X lens and a CoolSNAP digital camera.

### Quantification of Rbfox1-positive cells and RGCs

The retinas were dissected from the eyeballs, fixed in 4% paraformaldehyde in 0.1 M phosphate buffer and incubated with 10% serum for 1 hour to block nonspecific staining. The retinas were incubated with the primary antibodies overnight at 4°C. After washing, the retinas were incubated with corresponding secondary antibodies overnight at 4°C. The retinas were mounted flat with several radial cuts on a glass slides with the GCL facing upward. Quantitative analysis of Rbfox1-expressing cells in the GCL was performed by counting Rbpms-, calbindin-, Rbfox2-, Rbfox1/Rbpms-, Rbfox1/calbindin- and Rbfox1/Rbfox2-positive cells. The retinas were divided into four quadrants: superior, inferior, nasal and temporal and four sampling fields (0.31 x 0.31 mm each) were imaged at 0.5 mm from the center of the optic nerve in each retina. Data are presented as the mean ± SEM. Four retinas were used for each immunostaining.

Quantification of RGCs was performed as previously described [41]. Following immunolabeling with Rbpms antibodies, the retinas were divided into superior, inferior, nasal, and temporal quadrants and four sampling fields (0.32 × 0.24 mm each) were imaged at each region of 0.5, 1, 1.5, and 2 mm from the center of the optic nerve in each retinal quadrant. Quantification was performed in a masked manner. Data are presented as the mean ± SEM. Retinas from three control and three Rbfox1 KO animals were used in these experiments.

### Behavioral test

A visual cliff test was used to access visual function [27]. A chamber with a glass bottom (dimensions 16 × 20 inches) was placed on the edge of the table so its one half was resting on the table surface (“shallow” side) and the second half was suspended above the floor (the “cliff drop” or “deep side”). The table and the floor were covered with 1-inch squared black checker linoleum. The apparatus was placed under a double fluorescent strip light which provided equal illumination of both sides of the glass surfaces. Three homozygous *Rbfox1*^loxP/loxP^ mice and three homozygous *Rbfox1*^loxP/loxP^; UBC-Cre^+/-^ mice (4 months of age) were used. The vibrissae were removed to eliminate tactile placing responses that could interfere with testing of visual function. Ten trials were given to each mouse. One set of trials consisted of placing the mouse on the shallow side and recording the time the animal spent on the deep side during 5 minutes of testing. The other set of trials consisted of placing the mouse on the center platform (a ridge of aluminum ½ inches high and 1-inch-wide) of the visual cliff apparatus and recording the side on which the mouse stepped. The glass and central platform were thoroughly cleaned after each test. Five control and five *Rbfox1* KO animals were used in these experiments. Data are presented as the mean ± SD of ten independent experiments.

### RNA sequencing (RNA-seq) analysis of *Rbfox1* KO retinal transcriptome

Total retinal RNA from 3 Rbfox1 KO and 3 control animals were isolated with an RNeasy mini kit (Qiagen, Germantown, MD). Total RNA was processed at the UCLA Neuroscience Genomics Core (www.semel.ucla.edu/ungc) with Ribo-Zero Gold kit (Epicentre, WI) to remove ribosomal RNAs. Sequencing libraries were prepared using the Nugen Ovation sample prep kit following manufacturer's protocol. After library preparation, amplified double-stranded cDNA was fragmented into 125 bp (Covaris-S2, Woburn, MA) DNA fragments, which were (200 ng) end-repaired to generate blunt ends with 5’- phosphates and 3’- hydroxyls and adapters ligated. The purified cDNA library products were evaluated using the Agilent Bioanalyzer (Santa Rosa, CA) and diluted to 10 nM for cluster generation in situ on the HiSeq paired-end flow cell using the CBot automated cluster generation system. All samples were multiplexed into a single pool in order to avoid batch effects [42] and sequenced using an Illumina HiSeq4000 sequencer (Illumina, San Diego, CA) at 75bp-paired-end sequencing, yielding between 49 and 78 million reads per sample. Quality control was performed on base qualities and nucleotide composition of sequences. Alignment to the M. musculus (mm10) refSeq (refFlat) reference gene annotation was performed using the STAR spliced read aligner with default parameters [43]. Additional QC was performed after the alignment to examine: the level of mismatch rate, mapping rate to the whole genome, repeats, chromosomes, key transcriptomic regions (exons, introns, UTRs, genes), insert sizes, AT/GC dropout, transcript coverage and GC bias. One experimental sample had poor mapping rate (48%) and was excluded from further analyses. The final dataset consisted of 2 experimental samples and 3 controls. Total counts of read-fragments aligned to candidate gene regions were derived using HTSeq program (www.huber.embl.de/users/anders/HTSeq/doc/overview.html) with mouse mm10 (Dec.2011) refSeq (refFlat table) as a reference and used as a basis for the quantification of gene expression. Between 72 and 86% (average 82.7%) of the reads mapped uniquely to the mouse genome. Only uniquely mapped reads were used for subsequent analyses. Differential expression analysis was conducted with R-project and the Bioconductor package edgeR [44]. Statistical significance of the differential expression was determined at false discovery rate (FDR) <0.1. The Functional Annotation Tool of the DAVID Bioinformatics Resources 6.8 (https://david.ncifcrf.gov/) was used to analyze RNA-seq data with focus on genes that are known to have neuron-specific functions and KEGG (Kyoto Encyclopedia of Genes and Genomes) DISEASE Database was used to identify genes associated with various forms of neurological conditions. RNAseq data has been deposited within the Gene Expression Omnibus (GEO) repository (http://www.ncbi.nlm.nih.gov/geo), accession number GSE105084.

### Quantitative real-time PCR

Total RNA was extracted from mouse retinas using RNeasy mini kit (Qiagen) according to the manufacturer’s instructions. First-strand cDNA was synthesized with SuperScript III First-Strand Synthesis System (Thermo Fisher Scientific). PCR was carried out with the SYBR Green PCR Master Mix (Applied Biosystems/Life Technologies, USA) in a total volume of 10 μL Primers for real-time PCR are listed in Table 3. The LightCycler 480 II (Roche Applied Science, Mannheim, Germany) instrument was used for amplification and real-time quantitative detection of PCR products. Target genes expression levels were normalized to the threshold cycle (Ct) of mouse GAPDH. The expression level of each gene was calculated relative to the expression of the control group: 2-ΔΔCt, where ΔΔCt = Exp (Ct, target–Ct, GAPDH)–Ctrl (Ct, target–Ct, GAPDH). The data are shown as the mean ± SEM of three replicates.

**Table 3:** Primers used for quantitative real-time PCR.

Gene	Primer sequence (5′-3′)	
Ptgds	F:ACGCAGGTGAGAGAAGTCAG	R:GCTGGGATCTTGAGAGTGACA
Vamp1	F:TAGAAATGCTGCTCGTGGTCC	R:CCATGGACACAAATACCCTTC
Rgr	F:AGGTCAGAGGTCAGGAAGATG	R:TGGGTCCAACATAAGGTTAGACT
Calb1	F:CCACAACCACTTGCTAGTGATAC	R:GTGCCACACATCCTGAATAGTTC
Trpv1	F:AGGATCTACTCAGGGAGCAATAG	R:CATGGTTAGATTCACAGCTCGC
Esr1	F:GACTAGCAGAATTCTGTCTCC	R:TACCAAGATTTAGCCCTGGAATC
Oacyl	F:CTTGGAACGTGAATCTGATTTTG	R:GAAAGACCATGACTGTGTGCCT
Syce1	F:CTGTCTCTTGCTCACCACTAG	R:GGAACTAAATTACCAAGTGGCTT

### Statistical analysis

For RGC quantification, an unpaired Student’s t-test was used. P < 0.05 was considered statistically significant. For the behavior test, a repeated measures ANOVA model was used to analyze the mean difference between the two groups in time spent on the deep side after controlling for repeated measurements within each animal. A logistic regression model with generalizing estimating equation (GEE) was used to analyze the preference of animal from the two groups to step down on the shallow side after controlling for repeated measurements within each animal.

## Supporting information

S1 Table Differentially expressed genes in Rbfox1 KO animals. A complete gene list analyzed by RNA sequencing, their fold changes (Rbfox1 KO vs. control), uncorrected and FDR corrected p-values, and absolute gene level quantification using FPKM and log count per million are presented.
(XLSX)

S1 Fig Top rated differentially spliced genes in Rbfox1 KO animals. (PDF)
