# Supplementary material for: Downregulation of splicing regulator RBFOX1 compromises visual depth perception
Source: PLoS One. 2018 Jul 12;13(7):e0200417. doi: 10.1371/journal.pone.0200417 (PMC6042722; doi:10.1371/journal.pone.0200417)
Supplement: S1 Fig — (PDF) [file pone.0200417.s002.PDF]

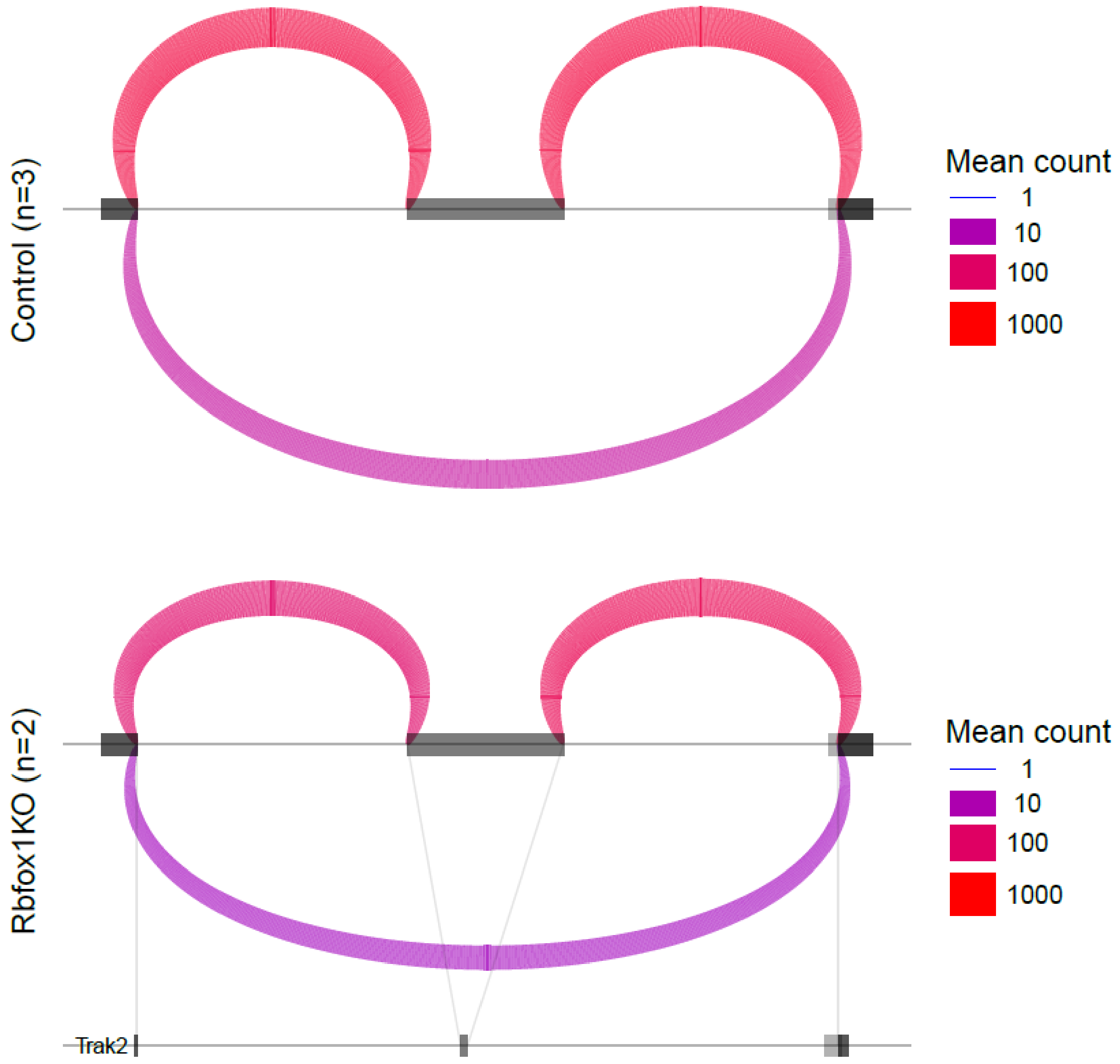

Control (n=3)
Mean count
1
10
100
1000
Rbfox1KO (n=2)
Mean count
1
10
100
1000
Trak2

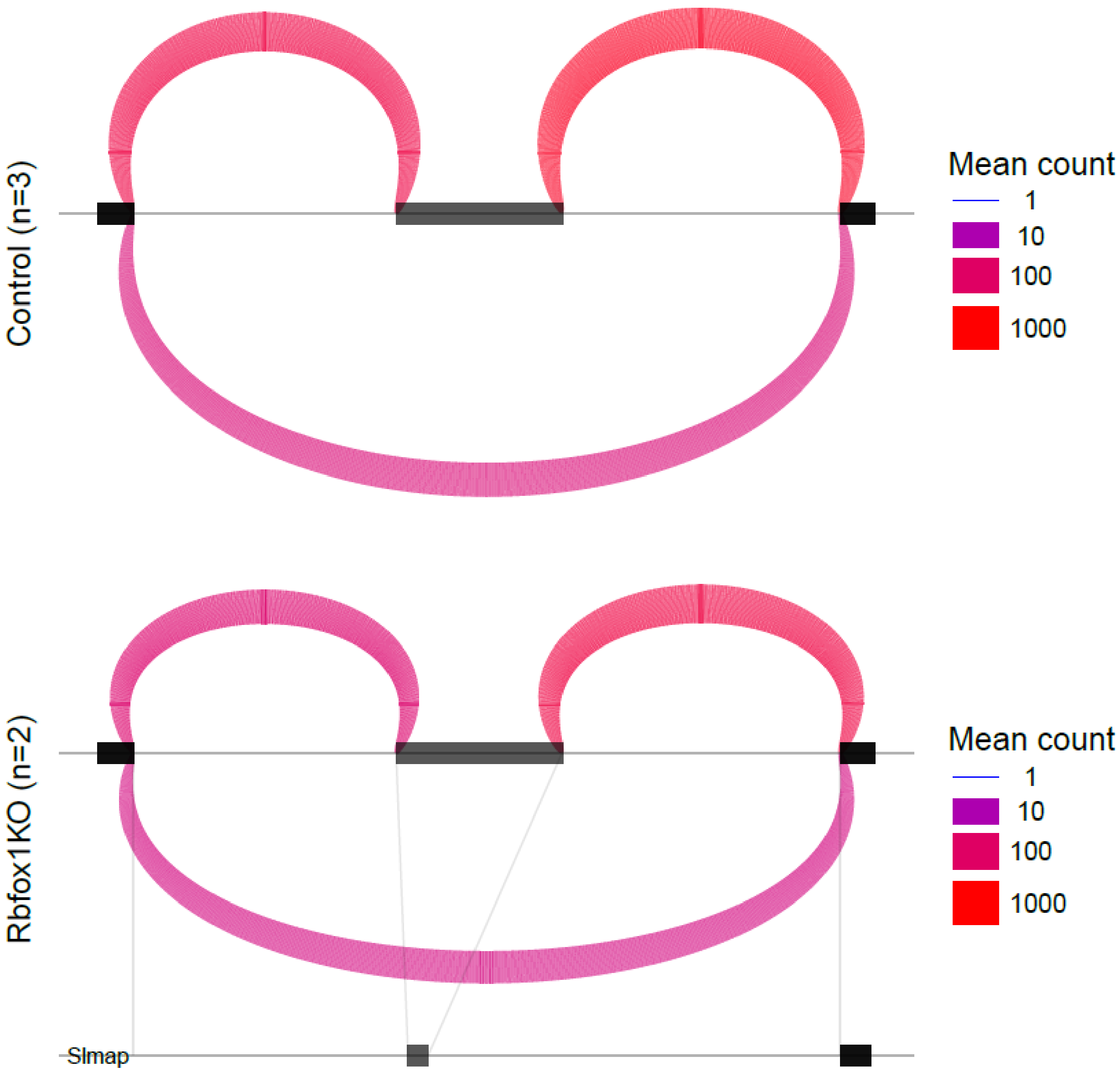

Control (n=3)
Mean count
1
10
100
1000
Rbfox1KO (n=2)
Mean count
1
10
100
1000
Slmap

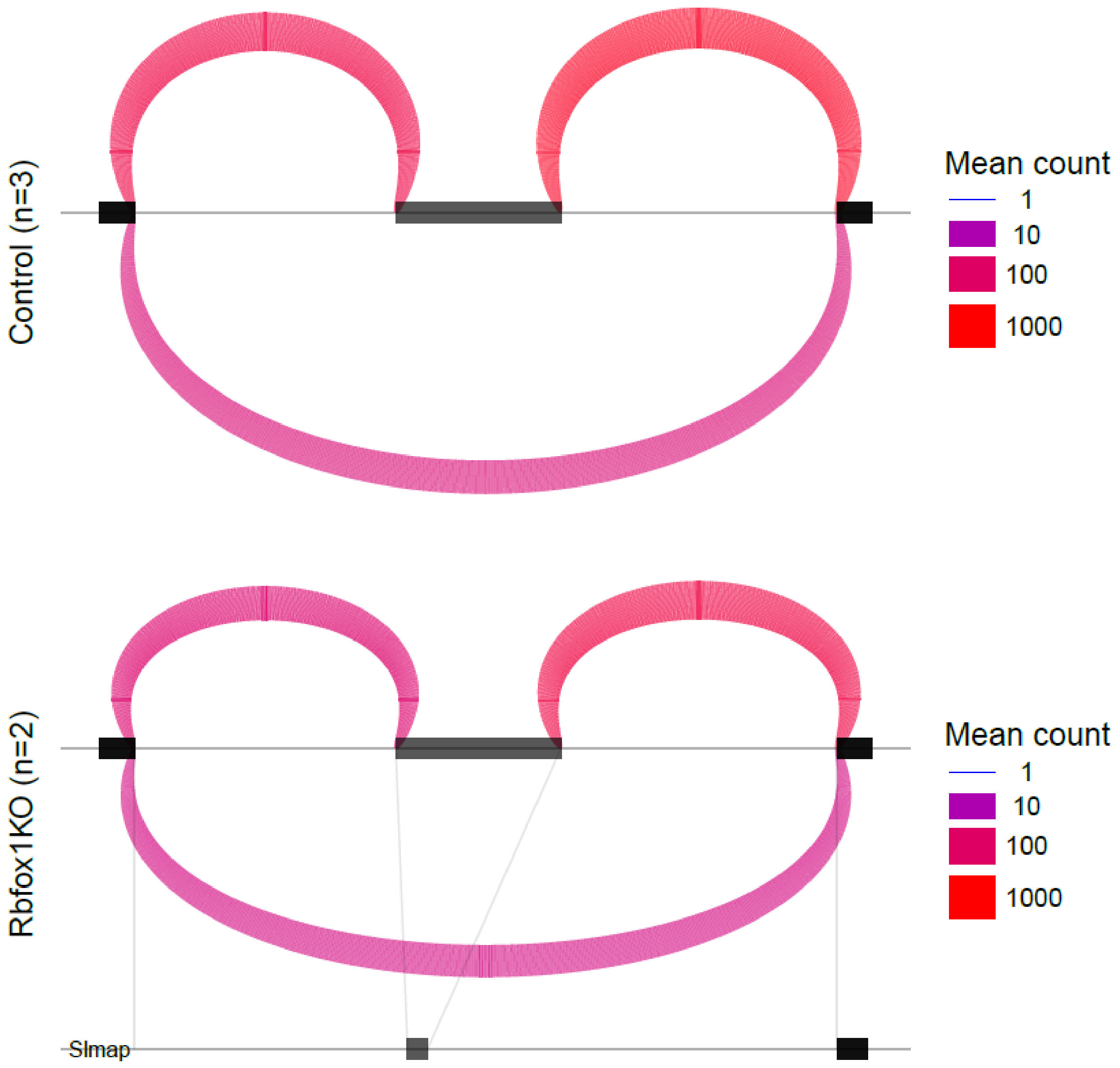
Control (n=3)
Mean count
1
10
100
1000
Rbfox1KO (n=2)
Mean count
1
10
100
1000
Slmap

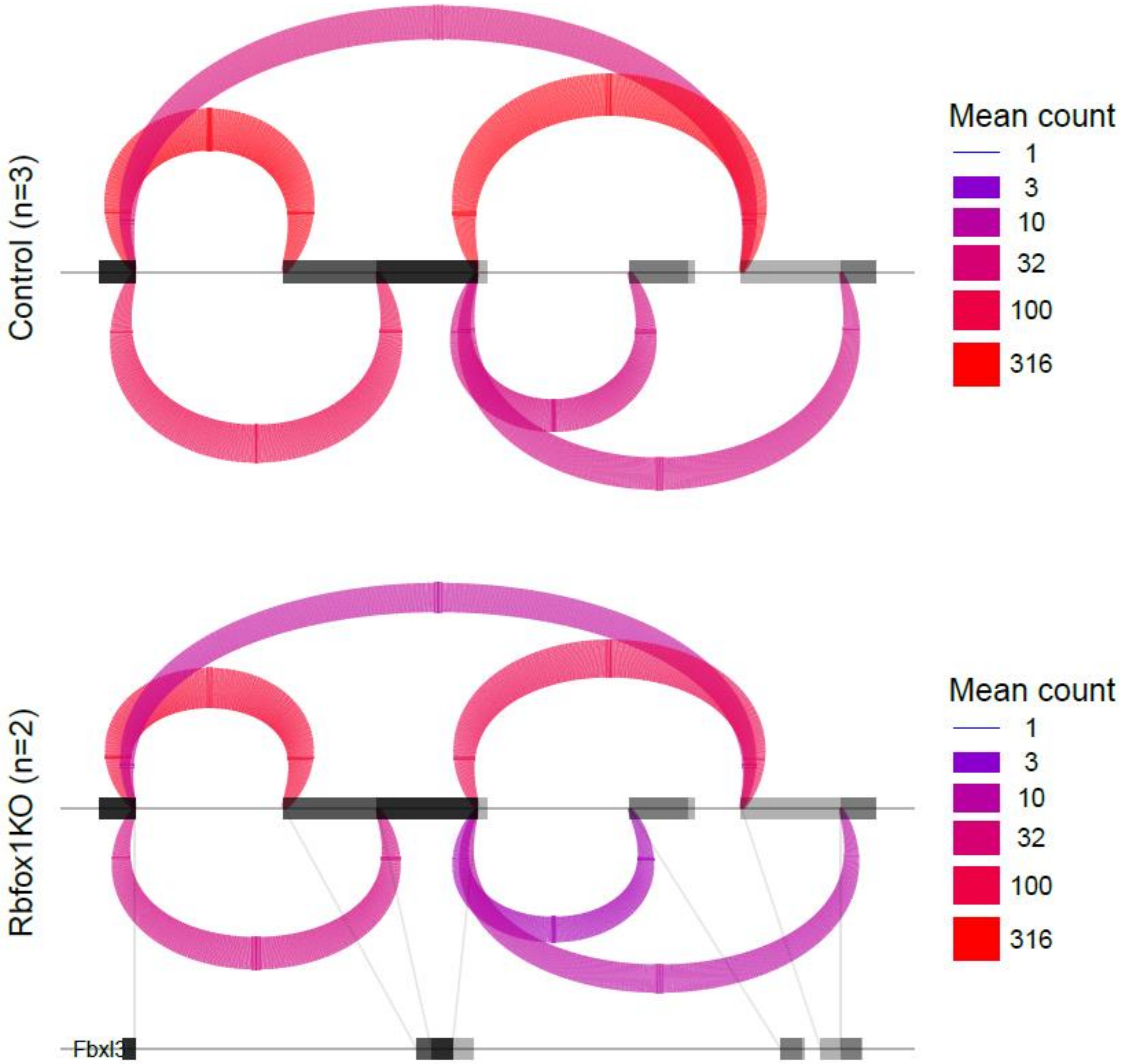

Control (n=3)
Mean count
1
3
10
32
100
316
Rbfox1KO (n=2)
Mean count
1
3
10
32
100
316
Fbxl3

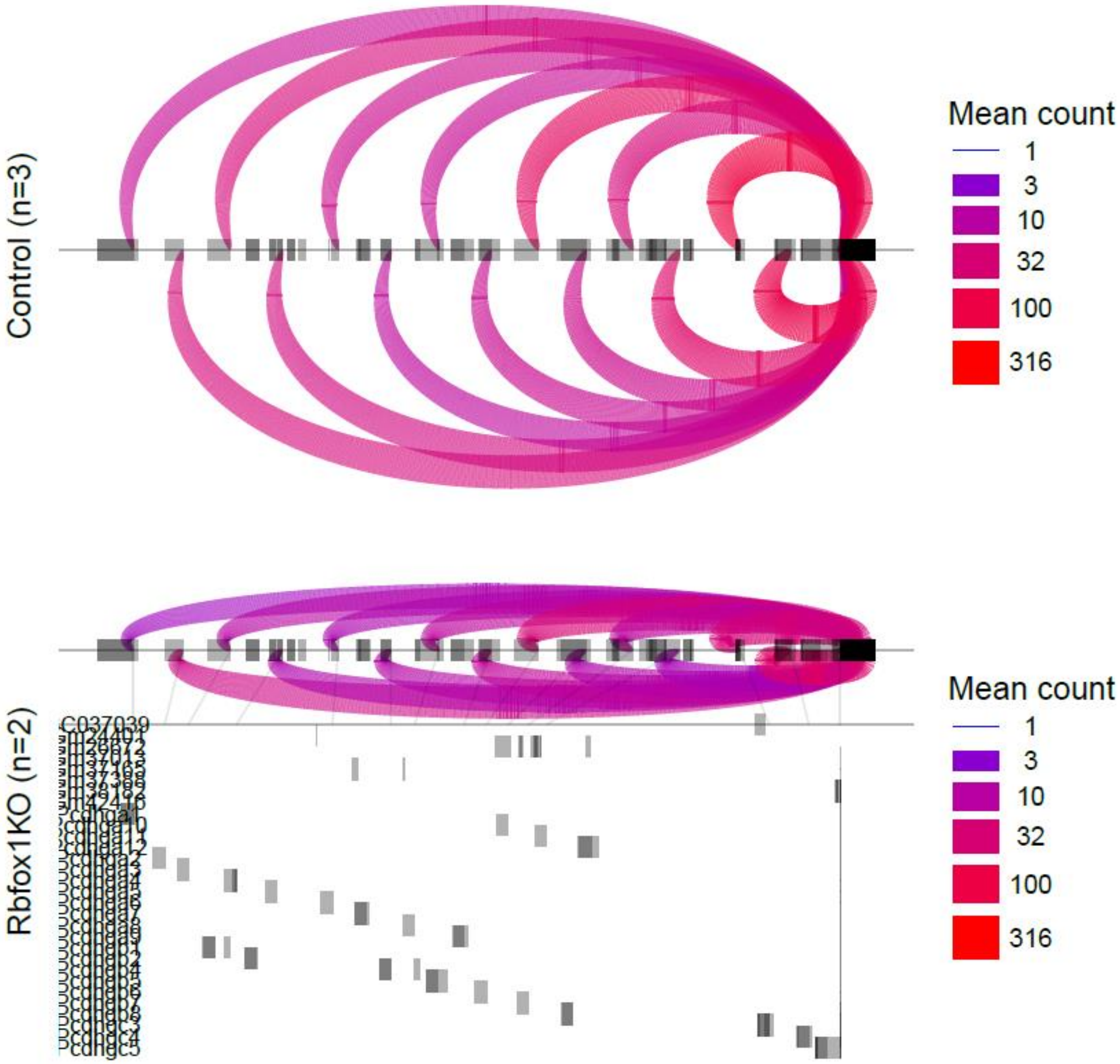

Control (n=3)
Mean count
1
3
10
32
100
316
Rbfox1KO (n=2)
Mean count
1
3
10
32
100
316

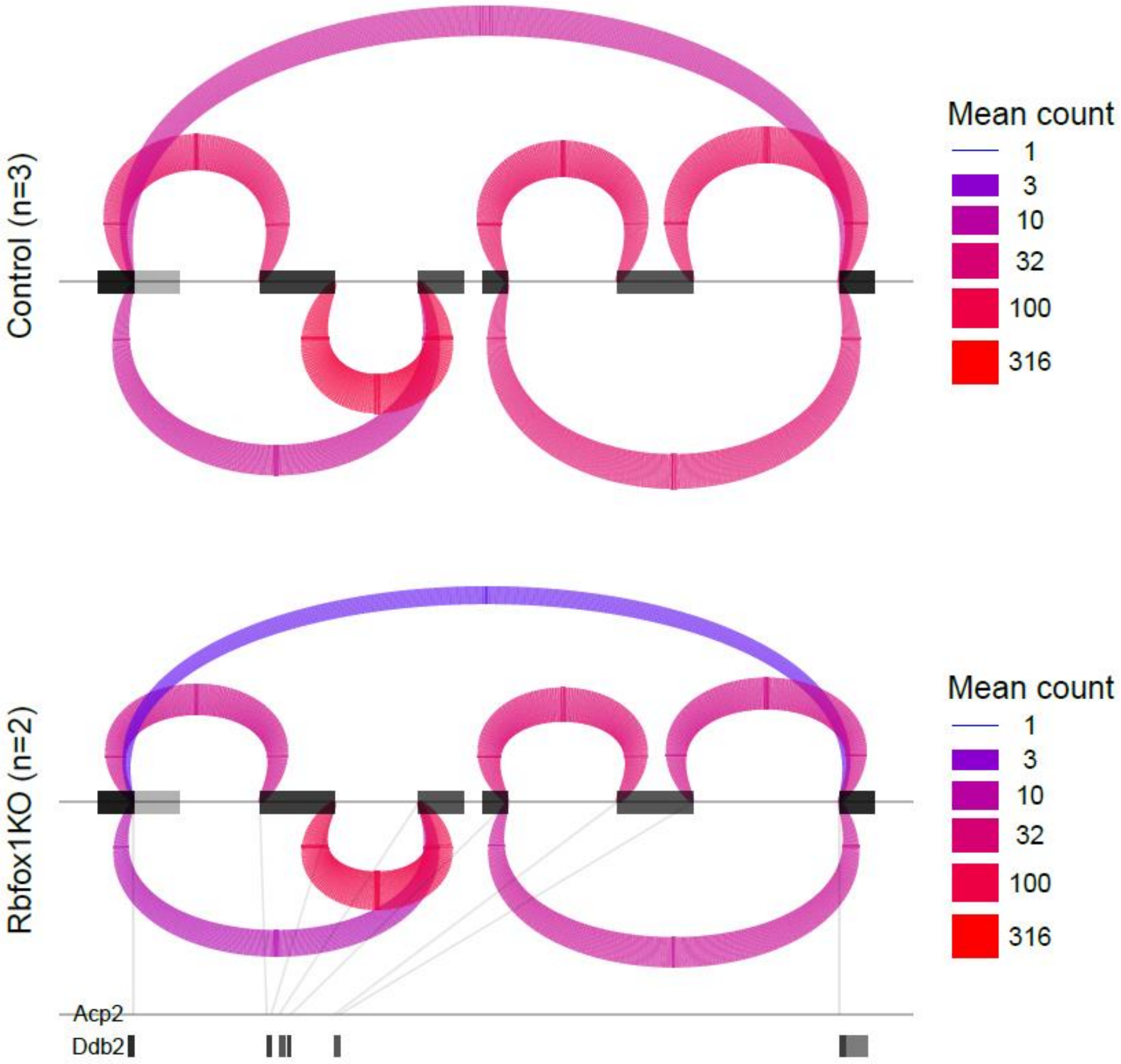
Control (n=3)
Mean count
1
3
10
32
100
316
Rbfox1KO (n=2)
Mean count
1
3
10
32
100
316
Acp2
Ddb2

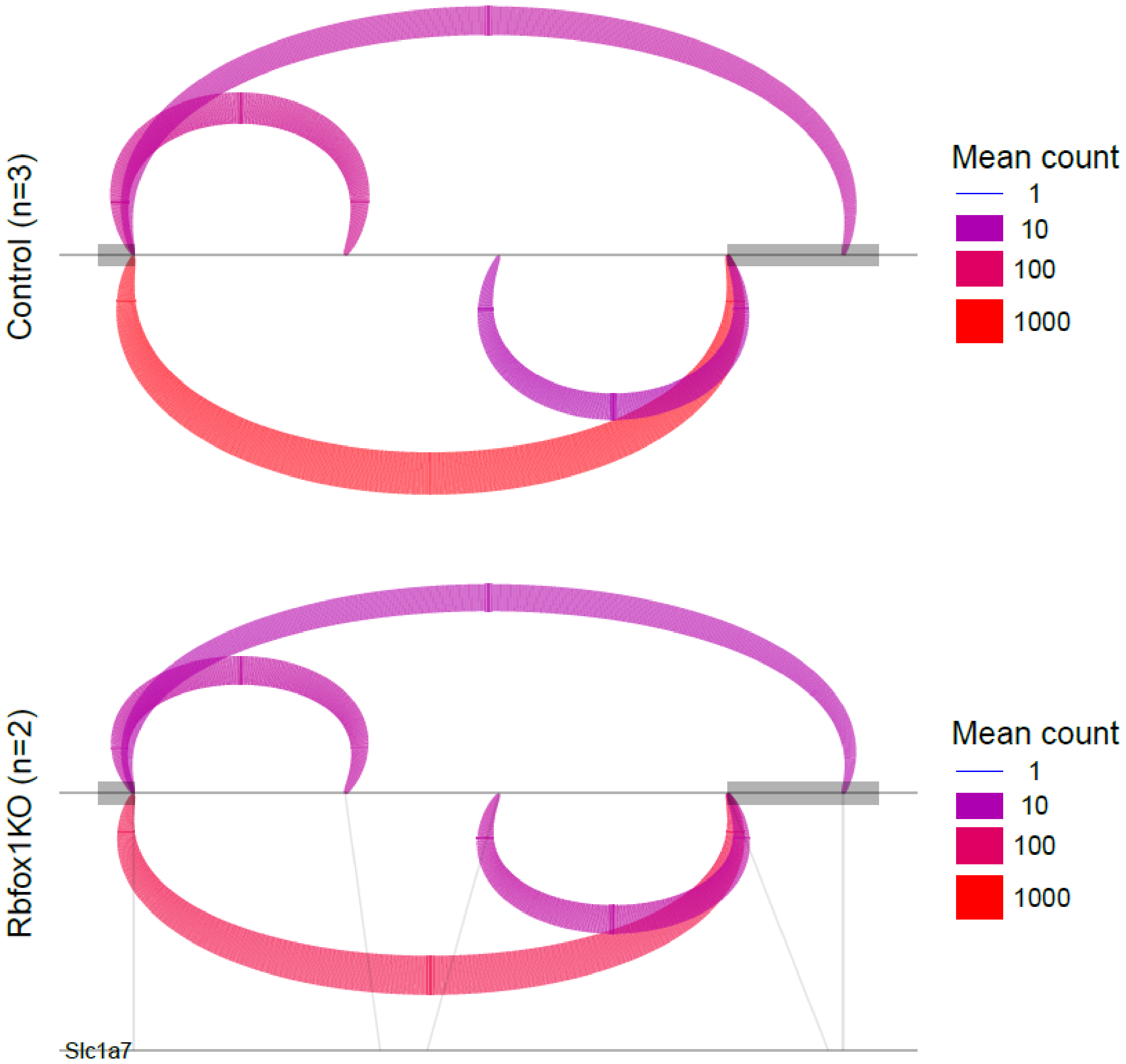
Control (n=3)
Mean count
1
10
100
1000
Rbfox1KO (n=2)
Mean count
1
10
100
1000
Slc1a7

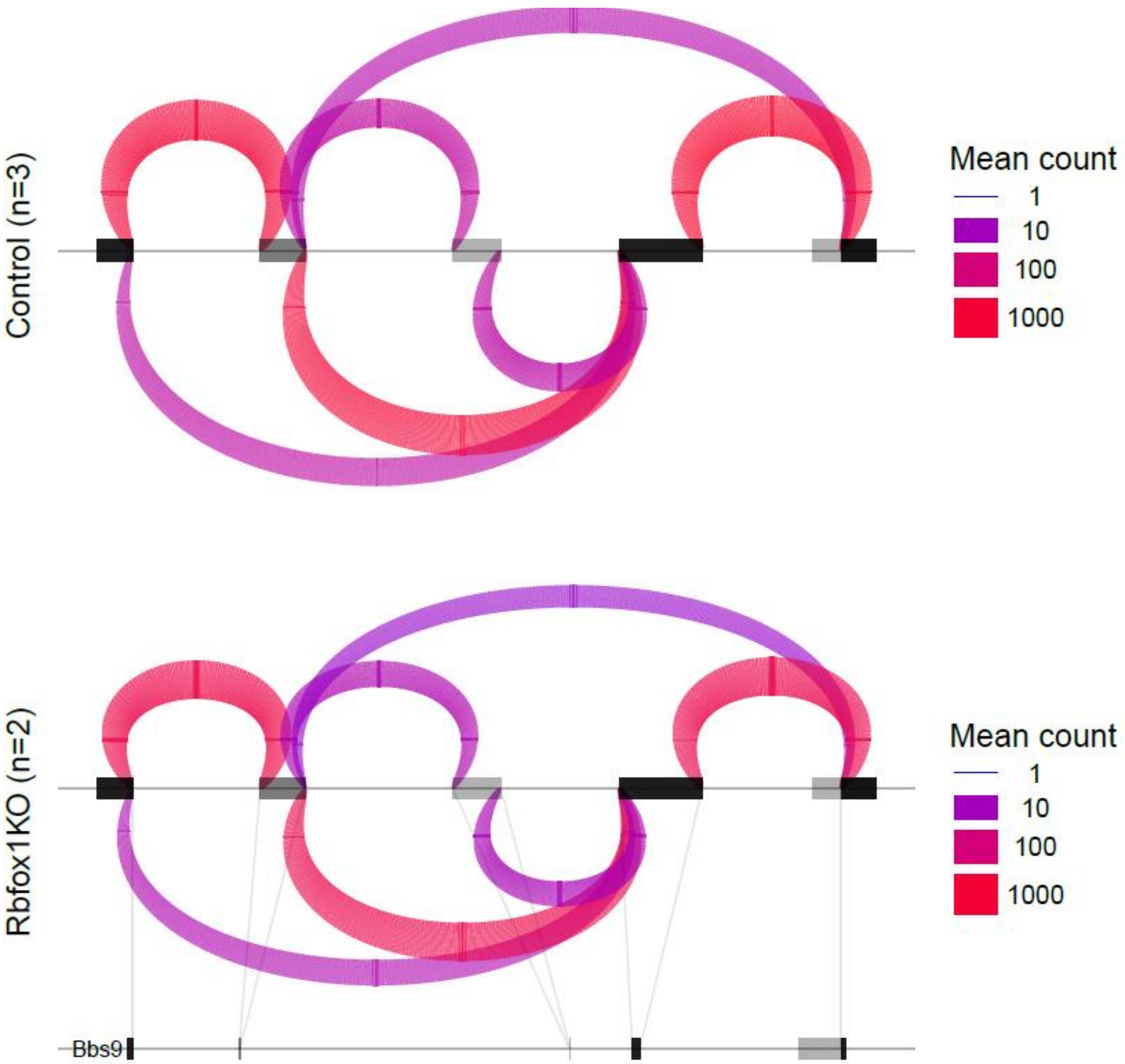

Figure 1S. Top rated differentially spliced genes in Rbfox1 KO animals.
